# A data‐driven sliding‐window pairwise comparative approach for the estimation of transmission fitness of SARS‐CoV‐2 variants and construction of the evolution fitness landscape

**DOI:** 10.1002/qub2.70003

**Published:** 2025-04-21

**Authors:** Md Jubair Pantho, Richard Annan, Landen Alexander Bauder, Sophia Huang, Letu Qingge, Hong Qin

**Affiliations:** ^1^ Department of Computer Science and Engineering University of Tennessee at Chattanooga Chattanooga Tennessee USA; ^2^ Department of Computer Science North Carolina Agricultural and Technical State University Greensboro North Carolina USA; ^3^ Thomas Jefferson High School for Science and Technology Alexandria Virginia USA; ^4^ School of Data Science Department of Computer Science Old Dominion University Norfolk Virginia USA

**Keywords:** fitness landscape, pairwise comparative estimation, SARS‐CoV‐2, sliding‐windows, viral variant fitness

## Abstract

Estimating the transmission fitness of SARS‐CoV‐2 variants and understanding their evolutionary fitness trends are important for epidemiological forecasting. Existing methods are often constrained by their parametric natures and do not satisfactorily align with the observations during COVID‐19. Here, we introduce a sliding‐window data‐driven pairwise comparison method, the differential population growth rate (DPGR) that uses viral strains as internal controls to mitigate sampling biases. DPGR is applicable in time windows in which the logarithmic ratio of two variant subpopulations is approximately linear. We apply DPGR to genomic surveillance data and focus on variants of concern (VOCs) in multiple countries and regions. We found that the log‐linear assumption of DPGR can be reliably found within appropriate time windows in many areas. We show that DPGR estimates of VOCs align well with regional empirical observations in different countries. We show that DPGR estimates agree with another method for estimating pathogenic transmission. Furthermore, DPGR allowed us to construct viral relative fitness landscapes that capture the shifting trends of SARS‐CoV‐2 evolution, reflecting the relative changes of transmission traits for key genotypic changes represented by major variants. The straightforward log‐linear regression approach of DPGR may also facilitate its easy adoption. This study shows that DPGR is a promising new tool in our repertoire for addressing future pandemics.

## 1 INTRODUCTION

The COVID‐19 pandemic caused by the severe acute respiratory syndrome coronavirus 2 (SARS‐CoV‐2) has underscored the importance of estimating transmission fitness for variants to predict viral evolutionary dynamics. Since December 2019, SARS‐CoV‐2 has undergone many mutations and likely rounds of recombination, leading to the emergence of multiple variants with different levels of transmissibility and virulence [1, 2, 3]. Some variants became more dominant during the transmission and were labeled ‘variants of concern’ by Greek letters such as Alpha, Delta, and Omicron [4, 5, 6].

The basic reproductive number *R*_0_ is often used to gauge viral transmission, representing the average number of new infections caused by an infected individual in a susceptible population [7]. *R*_0_ is often derived from the SIR (susceptible–infectious–removed) model and its many derivations. In practice, the effective reproductive number, *R*_*t*_, is often used for a real‐time indicator that estimates the average number of secondary infections caused by an infectious individual in a population over time [8, 9]. Both *R*_0_ and *R*_*t*_ are estimated from incidence data that often lack information on particular variants. Consequently, it is typically challenging to use *R*_0_ and R_*t*_ to differentiate the transmission fitness of emerging variants.

Phylogenetic analysis, as a classic evolutionary approach, was used to understand the evolutionary fitness changes of SARS‐CoV‐2 based on its mutations [3, 10, 11]. However, phylogenetic approach has limited usage for SARS‐CoV‐2 because of the low genetic variability among SARS‐CoV‐2 sequences, root placement uncertainty, recurrent mutations, recombination among subvariants, and geographic and temporal biases [10, 11, 12]. The limitation of phylogenetic analysis was highlighted by the unexpected evolutionary pattern of the Omicron variant [13].

Multinomial or hierarchical logistic regressions implemented with softmax are recent approaches to estimating the fitness of variants of SARS‐CoV‐2 [14, 15, 16, 17]. Multinomial logistic regressions can be used in conjunction with a hierarchical Bayesian framework. The estimated variant fitness can be used for more accurate forecasts up to 30 days and to identify mutations associated with fitness gains.

Alternative approaches for variant fitness estimation can be complementary and address the limitations of existing methods. To this end, we present a sliding‐window data‐driven pairwise comparative approach to estimate relative fitness for two viral strains, the differential population growth rate (DPGR), based on straightforward log‐linear regression. DPGR is an additive distance, and its comparative nature makes it more tolerant to some sampling biases. We apply DPGR to estimate the transmission fitness of variants of SARS‐CoV‐2. We found the log‐linear assumption can hold in a wide range of time windows. We also constructed a fitness landscape for the evolving variants, and shed light on the recent evolution of SARS‐CoV‐2.

## 2 RESULTS

### 2.1 Differential population growth rate (DPGR) for pairwise relative viral transmission fitness estimation

Motivated by using an internal control to mitigate sampling biases often associated with SARS‐CoV‐2, we designed a pairwise comparative approach on sliding time windows, the differential population growth rate (DPGR), for estimating the pairwise transmission fitness advantages of the variants of SARS‐CoV‐2. DPGR is calculated by log‐transforming the ratio of the growth rate of two exponentially growing populations in the applicable time windows and taking the growth latencies into account, as shown by Equation (1).

(1) $$\log_{10}\left(\frac{N_{1}}{N_{2}}\right)=\left(g_{1}-g_{2}\right)t+C={\text{DPGR}}_{1,2}\cdot t+C$$

where *N*_1_ and *N*_2_ are two exponentially growing viral sub‐populations, *g*_1_ and *g*_2_ are the growth coefficients, the constant C incorporates the lag time of the growth of the two sub‐populations, and *t* represents time measured in days in this study. The log‐transformed ratio of the growth is a linear model where a positive slope indicates population *N*_1_ grows faster than population *N*_2,_ and a negative slope indicates the opposite. DPGR_1,2_ = (*g*_1_ − *g*_2_) is defined for variants 1 and 2 (detailed induction of DPGR is in the supporting information.) In practice, sliding time windows were applied, and the appropriate periods for the log‐linear approximation were selected based on linearity evaluation using *R* ^2^ and *p*‐value.

During COVID‐19, some variants dominate in different periods and are challenging to be co‐sampled at the same location in the same time window, especially given the limited genomic surveillance capacity. Based on the property of logarithms

$$\log_{10}(a/b)=\log_{10}(a/c)+\log_{10}(c/b),$$

we can find another variant *c* which can estimate DPGR_*a*,*b*_ through

$${\mathrm{DPGR}}_{a,b}={\mathrm{DPGR}}_{a,c}+{\mathrm{DPGR}}_{c,b}$$

as long as variant *c* can be co‐sampled with both variant *a* and *b* at the same location (detailed formulation in the supporting information). For instance, we estimated DPGR_Alpha,Delta_ through

$${\mathrm{DPGR}}_{\mathrm{Alpha},\mathrm{Delta}}={\mathrm{DPGR}}_{\mathrm{Alpha},\mathrm{Beta}}+{\mathrm{DPGR}}_{\mathrm{Beta},\mathrm{Delta}},$$

even though we had insufficient weekly co‐observations of Alpha and Delta variants in the same location in the genomic sampling data from GISAID [18].

### 2.2 Overview of the computational workflow

**FIGURE 1:**
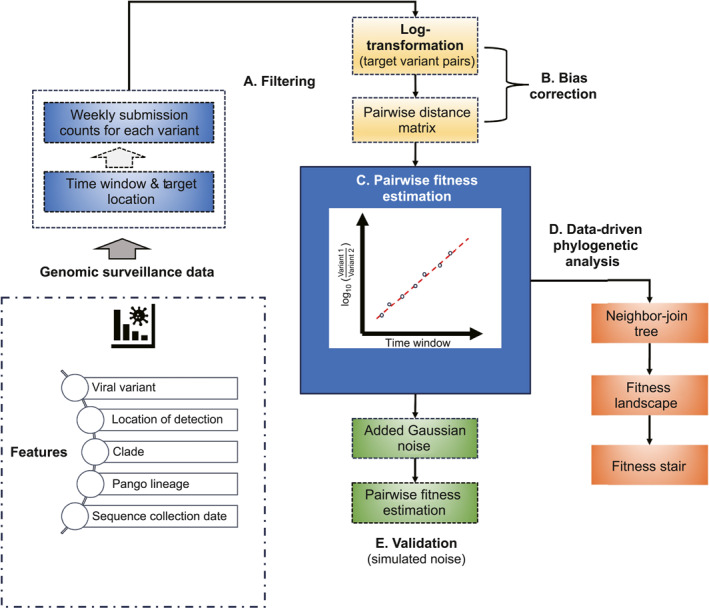
Overall computational workflow of the DPGR model for transmission fitness estimation.

As shown in Figure 1, we retrieved the genomic variant surveillance data for SARS‐CoV‐2 from GISAID up to 16 June 2022. The variants of concern (VOCs) in the retrieved data set include Alpha, Beta, Gamma, Delta, and Omicron. The GISAID surveillance data include collection dates and locations. We used the weekly occurrence of each VOC at selected locations and scanned for time windows in which DPGR linearity assumption is applicable. The DPGR‐applicable time windows were typically selected for at least 4 weeks and with a linear fit of *R* ^2^ value greater than 0.9. We applied DPGR to various countries and continents. For comparison, we estimated the transmission fitness for VOCs designated by the WHO and Pango lineage [19] provided by GISAID. Based on the pairwise fitness estimation, we constructed fitness stairs and landscapes to capture the shifting evolutionary trends of SARS‐CoV‐2 variants.

### 2.3 Country level viral transmission revealed by DPGR with the Omicron and Delta variants as an example

**FIGURE 2:**
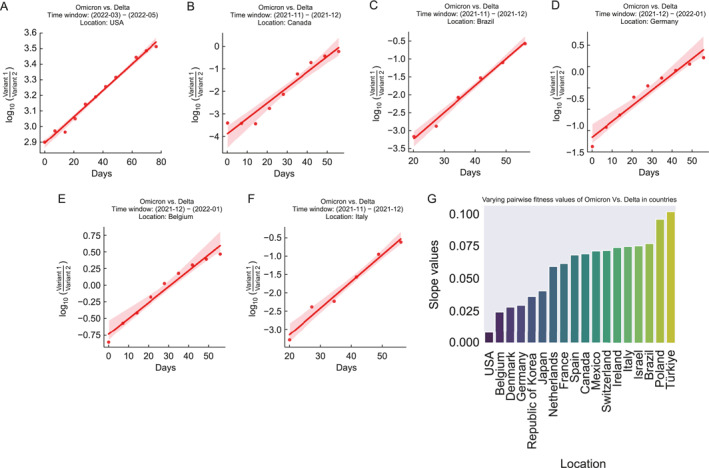
Pairwise transmission fitness estimation of the Omicron variant compared to the Delta variant in several countries. (A–F) Illustrates the sharp increase of pairwise transmission fitness of the Omicron variant compared to the Delta variant in the target countries. The *y*‐axis in the plots refers to the log‐transformed ratio of growth, and the *x*‐axis refers to the time window in days. (G) The bar plot visualizes the estimated transmission fitness values in all the analyzed regions.

We first present the utility of DPGR to estimate variant transmission fitness at the country level using the pair of Omicron and Delta variants as an example. We performed sliding time window analysis to select time windows in which the DPGR assumption held well. For illustration, estimations of DPGR between the Omicron and Delta variants in a few countries are presented in Figure 2. A set of target countries, including the United States, Canada, Brazil, Republic of Korea, Ireland, Denmark, the Netherlands, Italy, Türkiye, Belgium, Poland, Israel, Japan, Switzerland, Spain, France, Mexico, and Germany, are selected for showcasing the application of DPGR (Figure 2 and Figure S1). In the United States, within the time window from March 2022 to May 2022, the logarithmic values of the Omicron versus Delta population fit with a linear model (Figure 2A) with an *R* ^2^ value of 0.99 (Table S1 for the estimated slope and *R* ^2^ values for the aforementioned target countries). Applying DPGR in the selected countries yields a range of estimation of the Omicron versus Delta variants, termed DPGR_Omincron,Delta_ (Figure 2G), ranging from 0.008 to 0.1 with the average at 0.06. Among the analyzed countries, the highest DPGR_Omincron,Delta_ is observed in Türkiye (0.1) and the lowest in the United States (0.008). It can be observed that (Figure 2G) some European bordering countries (Denmark, Germany, Belgium, etc.) exhibited similar DPGR_Omincron,Delta_, indicating that these bordering countries share similar trends of SARS‐CoV‐2 epidemic transmission based on DPGR estimates.

Additionally, we fit DPGR to the Pango lineage labels GRA and GK that correspond to the WHO labels of the Omicron and Delta variants (Figure S3). The results of DPGR_GRA,GK_ are nearly identical to those of DPGR_Omicron,Delta_. The results here demonstrate the generalizability of DPGR. The similarity between DPGR WHO and GISAID labels is further illustrated in heatmaps (Figures S2 and S4). DPGR_GRA,GK_ estimate is 0.0094 in the United States and 0.1021 in Türkiye, with *R* ^2^ values of 0.99 and 0.97, respectively, that are consistent with DPGR_Omicron,Delta_ (Table S4).

Overall, the results here showed that DPGR is useful for examining the country‐level variations in viral transmissions which might reflect differences in regional responses, vaccine efficacies, population demographics, immune responses, environmental factors, and social and cultural factors.

### 2.4 Continent level viral transmission revealed by DPGR with the Omicron versus Delta variants as an example

**FIGURE 3:**
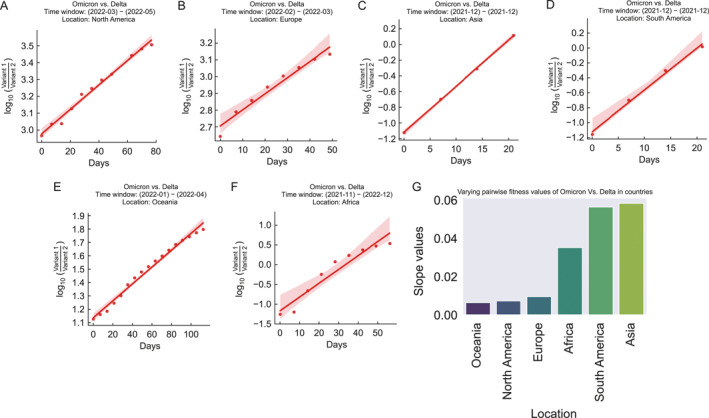
Pairwise transmission fitness estimation of the Omicron variant compared to the Delta variant in several continents. (A–G) Visualize the sharp increase of transmission fitness of the Omicron variant compared to the Delta variant in several continents. (G) Bar plots to visualize the fitness values in each continent.

To further examine the utility of DPGR, we applied it at the continental level using the Omicron versus Delta variants as an example. We preprocessed the data to weekly frequency for each variant in each continent. Figure 3A–G illustrates a positive rise in transmission fitness advantage of the Omicron variants in several continents in various time windows that indicates the geographic mobility of the circulating variant of concern. The DPGR estimates range from 0.006 to 0.058 with an average value of 0.03. The lowest relative transmission fitness is observed in Oceania (0.006) and the highest (0.058) in Asia. The *R* ^2^ values range from 0.92 to 0.99 (Table S1).

Similarly, we estimate DPGR for the corresponding GISAID labels GRA and GK (Figure S5) which show nearly identical trends. The pairwise transmission values between the variant pairs (GRA, GK, GRY, and GH) are illustrated as heat maps in Figure S6. The GRA variant demonstrates a relative fitness advantage, particularly in Asia and South America, with the smallest increase in Oceania. The range of the transmission fitness values between the comparison, DPGR_GRA,GK_, in several continents is 0.0064 (Oceania) to 0.0577 (Asia) with *R* ^2^ values 0.99 (Oceania) and 0.99 (Asia), respectively. The estimate between DPGR_GRA,GK_ indicates analogous estimated values as DPGR_Omicron,Delta_ estimates for the continents (Table S4).

Overall, the results here show applying DPGR at different geographic scales can reveal the regional heterogeneity of viral transmission and aid our understanding of the spread and impact of different SARS‐CoV‐2 variants.

### 2.5 Transmission variations of Omicron sublineages revealed by DPGR

To further examine the utility of DPGR, we applied DPGR in evaluating the sublineages of the Omicron variant [20, 21]. From the Pango lineage of the GISAID dataset, we selected the sublineage from BA.1 to BA.5 associated with the Omicron variant, using the weekly frequency for each sublineage at a location of interest, and selected the time windows where DPGR linearity held. In North America, the *R* ^2^ values for DPGR of BA.5 to other sublineages range from 0.931 to 0.993 (Table S2).

**FIGURE 4:**
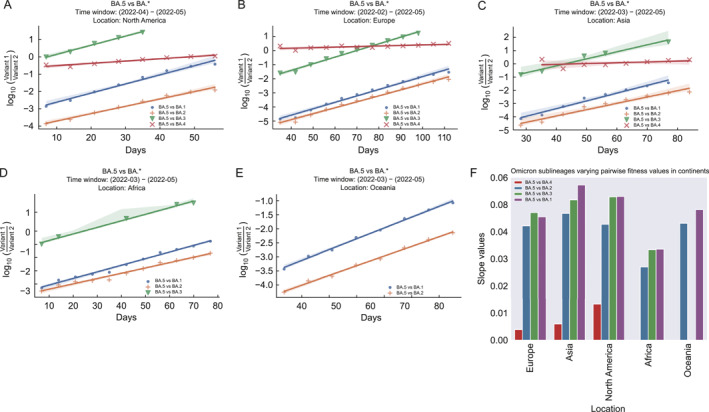
Pairwise transmission fitness estimation of the Omicron variant sub‐lineages in target continents. (A–E) Illustrates the estimated pairwise transmission fitness of the Omicron variant sub‐lineages (BA.5 with others sublineage). (F) Estimated fitness values in several continents.

For the sake of illustration, we chose the dominant sublineage BA.5 to compare with the other circulating sublineages (BA.1, BA.2, BA.3, and BA.4). Figure 4A–E) shows the pairwise DPGR estimation plots of BA.5 with other sublineages in five continents in time windows ranging from 5 weeks to 11 weeks in 2022. The linear fit of DPGR for the Omicron sublineages was often more pronounced than those in comparisons of major variants such as Omicron and Delta (Figures 2 and 3). The DPGR analysis demonstrates that the Omicron sublineage BA.5 consistently dominated over other sublineages though at varying levels of relative fitness compared to other sublineages (BA.1, BA.2, BA.3, and BA.4) across different continents as summarized in Figure 4F. For example, in Asia, BA.5 was found to have the fastest differential growth advantages over others, whereas its growth advantage was relatively moderate in Africa. In Europe and Asia, the DPGR plots of BA.5 over BA.4 were nearly flat, indicating codominance during the period of the analysis.

### 2.6 The evolution fitness landscape of SARS‐CoV‐2 variants and sub‐variants

**FIGURE 5:**
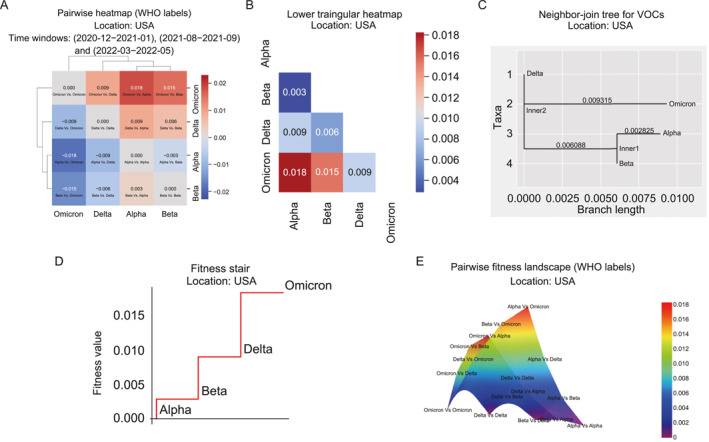
Progressive fitness evolution of variants of concerns (VOCs). (A) Pairwise heatmap of the transmission fitness for WHO variants in the United States. (B) Lower triangular heatmap with absolute pairwise distance to create the NJ tree, fitness stair, and fitness landscape. (C) Neighbor‐join tree. (D) Step plot visualizes the progressive transmission fitness gain of the WHO labeled variant of concerns (VOCs) in the United States. (E) Illustrates the pairwise transmission fitness landscape of the SARS‐CoV‐2 variants in the United States for the WHO labels. Variant pairs having higher pairwise transmission fitness is on the top of the landscape and vice‐versa.

The pairwise approach of DPGR offers an opportunity to capture the relative fitness landscape during the evolution of SARS‐CoV‐2 variants. At the time of our study, we had data to address the Alpha, Beta, Delta, and Omicron VOCs in the United States. All possible pairwise comparative analyses among these four VOCs lead to a 4 × 4 matrix (heatmap of Figure 5A). Examination by sliding windows shows that the DPGR model is applicable to the Alpha and Beta variants from March 2021 to April 2021, to the Beta and Delta variants from August to September 2021, and to the Delta and Omicron variants from March to May 2022 with corresponding *R* ^2^ values of 0.98, 0.97, and 0.98, respectively. For example, DPGR_omicron,beta_ = 0.015 and DPGR_beta,omicron_ = ‐ 0.015 which suggests that the Omicron variant outgrows the Beta variant by 1.5% daily (because e^0.015^ » 1.015). Some pairs of VOCs, such as Alpha and Delta, even without sufficient data points because of nonoverlapping periods, can be inferred based on the property of logarithms

$$\log_{10}(a/c)=\log_{10}(a/b)+\log_{10}(b/c)$$

(details in the supporting information), which enables us to compare transmission fitness for variants that dominate in different periods during the pandemic. For instance, we estimate

$${\mathrm{DPGR}}_{\mathrm{Alpha},\mathrm{Delta}}={\mathrm{DPGR}}_{\mathrm{Alpha},\mathrm{Beta}}+{\mathrm{DPGR}}_{\mathrm{Beta},\mathrm{Delta}},$$

even though we could not find sufficient weekly co‐observations of the Alpha and Delta variants in the same location in the genomic sampling data from GISAID.

It is worthy of emphasizing the DPGR is a relative measurement, and it is often more convenient to present the fitness on an ‘absolute’ scale. To do this, we first convert the DPGR matrix into a distance matrix (Figure 5B) by only focusing on the positive values. From the distance matrix, we apply the neighbor‐join tree [22] to capture the representative tree structure of fitness for the variants (Figure 5C). From the neighbor‐join tree, we infer the transmission fitness on a scale using the initial VOC Alpha as a reference point.

We inferred a fitness staircase (Figure 5D) depicting the transmission fitness gain of the VOCs—Alpha, Beta, Delta, and Omicron—in the United States. The progressive transmission fitness values for the Beta, Delta, and Omicron variants are shown relative to the initial variant, Alpha. The substantial increase in transmission fitness from the Delta to Omicron variants is clearly illustrated by the fitness staircase.

We constructed a neighbor‐join tree for the Omicron variant sub‐lineages (BA.1*, BA.2*, BA.3*, BA.4*, BA.5*) to gain a closer understanding of the progressive fitness evolution (Figure S11). We found that BA.5 has the fastest growth advantage with BA.4 as a close competitor. BA.2 and BA.4 have nearly similar fitness; however, BA.5 has rapidly increased transmission fitness than the other two sub‐lineages. BA.5 and BA.4 caused a rapid surge of cases in the United States likely because of their escape of neutralizing antibody [20, 21, 23].

We constructed a DPGR‐based fitness landscape for these circulating variants in the United States (Figure 5E) which illustrates the shifting evolutionary fitness changes among the variants during their evolution. The fitness landscape in evolutionary biology is used to visualize the relationship between genotypes and their evolutionary fitness [24, 25], and is of importance to understanding viral evolution [26, 27, 28, 29, 30, 31]. In Figure 5E, the variants represent the change of genotypes of SARS‐CoV‐2. The flying bird‐shaped landscape (Figure 5E) visualizes the pairwise fitness landscape of the WHO‐labeled variants of concern (VOCs). To generate the fitness landscape, we used the absolute pairwise DPGR. The pairs with the lowest fitness values are presented at the bottom of the landscape, and those with higher fitness climb progressively on the top of the hill. At the bottom of the hill, Omicron versus Omicron has zero fitness (from the estimated pairwise distance matrix), and as moved uphill, the Omicron variant exhibits significant fitness gain against the Delta, Beta, and Alpha variants. At the top of the hill, Omicron versus Alpha has the highest fitness gains as shown by the highest value of DPGR between the Omicron and Alpha variants. The Delta variant can be seen in the middle of the hill, reflecting its relative growth advantage between Omicron and other variants.

Overall, we show that the pairwise approach of DPGR can allow us to infer the fitness distance matrix fitness landscape, and fitness staircase through the neighbor‐join tree method.

### 2.7 Comparison of DPGR with other measurement

We compared the relative transmission fitness of SARS‐CoV‐2 variants using the DPGR model and the PyR_0_ model developed by another study [14]. We performed a linear correlation analysis (Figure S12) between the estimated DPGR and those by PyR_0_. We used the fold increase in relative fitness (R/RA) for the Alpha, Beta, Delta, and Omicron variants, relative to the Wuhan A strain, as provided in the ‘strains.tsv’ file from the supporting materials of the previous study. We log‐transformed (log_2_) the fold increase to make the values comparable with the DPGR model. We observed a strong linear correlation (*R* ^2^ = 1) between the estimates of PyR_0_ and DPGR. Interestingly, the requirement of a log transformation shows that the estimates of DPGR and PyR_0_ are on different scales. These analyses show that on one hand, DPGR gave a comparable estimate to the relative fitness of SARS‐CoV‐2 variants, and on the other hand, DPGR offers a different perspective to the previous method.

## 3 DISCUSSIONS

In this study, we present a data‐driven sliding‐window pairwise approach, DPGR, to capture the relative competitiveness of SARS‐CoV‐2 subpopulations and their changes in fitness during the pandemic. The pairwise nature of DPGR allows one subpopulation to serve as an internal control. The simplicity of log‐linear regression can facilitate the wide implementation and adoption of DPGR. The additive property of DPGR makes it suitable for distance matrix‐based analysis. The pairwise nature of DPGR enables the estimation of a relative fitness landscape.

The pairwise ratio approach can help reduce sampling errors that are nondiscriminatory to subvariants. Surveillance data on SARS‐CoV‐2 genomics are considerably underreported. It is reasonable to argue that most genomic surveillance methods are nondiscriminatory with respect to viral genomic sequences. Hence, the ratio between the two observed measurements would cancel out the nondiscriminatory sampling errors.

The DPGR model assumes that the logarithmic ratio of two viral variant subpopulations follows a linear trend within appropriately selected time windows. We conducted a sensitivity analysis of this DPGR log‐linear assumption with regard to parameter choices of sliding windows (Figure S14). The sensitivity analysis confirmed a robust log‐linear relationship that holds across a wide range of window sizes and start times. The analysis tested window sizes from 3 weeks to 25 weeks and starting weeks from late January to early July of 2022. The analysis found a large range of parameter combinations led to DPGR fit with *R* ^2^ values greater than 0.9 and *p*‐values less than 0.05.

We acknowledge that DPGR requires correctly labeled subpopulations. To avoid the negative value of DPGR, we recommend that the dominant variant is chosen to be the numerator part of DPGR.

There is a connection between DPGR and the classical logistical growth model proposed by Haldane [3]. DPGR can be considered as an extension of the logistic growth model log_10_(*p*_1_/(1 − *p*_1_)), in the sense that the denominator was replaced by the fraction of another subpopulation *p*_2_. There is also an interesting connection between DPGR and the softmax function that implemented the multivariate generalization of logistic growth [14]. The softmax function converts a set of real numbers into a probability distribution by exponentiating each value and dividing by the sum of these exponentials. The DPGR extends the exponential function with time‐lags, and gauges each subpopulation with another subpopulation.

Notably, DPGR can reveal regional differences in viral transmission. Regional differences in DPGR may be influenced by various factors. For example, age‐dependent infection rates of variants [32] and human population age structures could contribute to these regional differences. The differential dependence of variants on environmental factors, [33] such as humidity and temperature, is another possibility. Additionally, viral mutations [6, 34, 35], host genetic makeup [21, 36, 37, 38], immune responses [23, 36], and drug resistance [39] may play a role. Variations in vaccine efficacies are also a contributing factor.

Future studies of the DPGR model could explore its application beyond SARS‐CoV‐2 to other pathogens, integrate DPGR with genome‐wide association studies to identify key genetic mutations, and combine DPGR with machine learning to enhance predictive modeling.

## 4 CONCLUSION

Overall, we show that DPGR model offers a robust data‐driven approach for estimating the relative transmission fitness of SARS‐CoV‐2 variants. Through sliding‐window and pairwise comparisons, DPGR can effectively mitigate sampling biases and provides informative estimates across diverse temporal and geographical contexts. The additive property of DPGR enables the construction of fitness landscapes and distance matrices that can capture the dynamic evolutionary trends of viral variants. DPGR could facilitate future genome‐wide association studies to identify mutations associated with fitness changes in SARS‐CoV‐2 and interactions with host genetic factors. Our study suggests that DPGR is a promising tool in our repertoire for monitoring the transmission dynamics of pathogens.

## 5 MATERIALS AND METHODS

### 5.1 GISAID meta information

The dataset used for this research is provided by the global initiative on sharing all influenza data (GISAID) [18]. We download the metadata TSV file. There are a total of 18 columns in the dataset. We mostly focused on location, clade, Pango lineage, variant, and collection date. The variant column records the type of the SARS‐CoV‐2 variant according to the WHO (World Health Organization) proposed labels. Only variants of interest (VOIs) or variant of concern (VOCs) are labeled by the WHO.

Pango lineage is a dynamic nomenclature system that tracks the transmission and spread of the SARS‐CoV‐2 variants. Pango lineage uses phylogenetic diversity for naming the SARS‐CoV‐2 lineages that contribute most to the current propagation.

When a variant of newly emerged SARS‐CoV‐2 changes the pandemic dynamics and causes increased hospitalization and death, the World Health Organization (WHO) labels that specific variant as a variant of concern. The WHO also keeps the preventive measures on alert to check the further propagation. There are five VOCs labeled by the WHO, Alpha, Beta, Gamma, Delta, and Omicron, at the time of this study.

### 5.2 Data preprocessing

In the data preprocessing stage, we have taken some steps to ensure the data are in the correct format before passing it to the DPGR model. Initially, the raw dataset is loaded from a TSV (Tab‐separated value) file using libraries from pandas. Filtering of the rows is performed to ensure that the rows containing the non‐null ‘variant’ values are selected. We also mapped the variant name to a standardized format aligning with the GISAID or Pango nomenclature system to ensure consistency in variant names along the entire dataset. This ensured the uniformity of the naming of all the variants. Depending on the region of interest (country or continent), the ‘location’ column is mapped to the predefined lists of regions to facilitate country or continent‐level analysis. This standardization facilitated more accurate geographical analysis. Subsequently, the dataset was narrowed down to the most pertinent columns: ‘variant’, ‘location’, and ‘collection date’. The ‘collection date’ was then converted to a Python datetime format, and a new column representing the collection week was introduced to enable the weekly aggregation of the data.

The dataset is further filtered to a specified date range (‘2020‐01‐01’ to ‘2022‐05‐31′) and grouped by ‘variant’, ‘location’, and ‘date’ to obtain weekly frequency counts for a certain variant at a target location. Certain variants, which were less relevant for the analysis, were excluded to focus on the primary variants of interest such as Alpha, Beta, Gamma, Delta, and Omicron. The ‘date’ column was refined to extract the start date of each week, ensuring clarity in temporal analysis.

This weekly dataset was further summarized by summing the frequencies of each variant for each week and location, providing a clear and concise dataset for further analysis. This comprehensive preprocessing stage ensured the dataset was clean, standardized, and adequately prepared for detailed analysis and visualization, forming a solid foundation for understanding the spread and evolution of COVID‐19 variants across different regions and periods.

### 5.3 Sliding windows implementation

Typically, the ranges of the sliding time window were tested from 4 weeks to 12 weeks, considering both the *R* ^2^ and the *p*‐value of 0.05. For the region‐specific analysis, the time windows are aligned with the periods when the target variants are predominant in a region of interest.

### 5.4 Neighbor‐join tree

The neighbor‐joining method is an agglomerative clustering algorithm to create the phylogenetic tree of species. This method does not consider the constant rate of evolution; hence the branch length from each node to the tips varies. It takes in input as genome sequence data and calculates the distance between each pair of taxa. The neighbor‐joining algorithm uses the pairwise distance matrix to create an unresolved star‐shaped tree that is iterated over a few steps to find the tree’s branch lengths. From the distance matrix, it calculates a Q‐matrix and finds the distance between each pair of taxa. It joins the two taxa with a new node and connects with the central node. The Q‐matrix is calculated using this formula:

$$Q\left(i,j\right)=\left(n-2\right)d\left(i,j\right)-\sum_{k=1\;}^{n}d\left(i,k\right)-\sum_{k=1\;}^{n}d\left(j,k\right)$$

Then the algorithm calculates the distance of each of the taxa in the pair to the newly created node using the following equation:

$$\delta \left(f,u\right)=\frac{1}{2}d\left(f,g\right)+\frac{1}{2\left(n-2\right)}\left[\sum_{k=1}^{n}d\left(f,k\right)-\sum_{k=1}^{n}d\left(g,k\right)\right]$$

δ(g,u)=d(f,g)−δ(f,u)

Here, *u* is the newly created node, and *f*, *g* is the taxa in the pair. The distance from the other taxa from the newly created node is calculated using the following equation:

$$\delta \left(u,k\right)=\frac{1}{2}\left[d\left(f,k\right)-d\left(f,g\right)\right]$$

Here, *k* denotes the node to calculate the distance, and *u* refers to the newly created node.

### 5.5 Distance matrices

To construct the fitness stair and transmission fitness landscape, we created a pairwise distance matrix leveraging the DPGR method to infer pairwise transmission fitness between SARS‐CoV‐2 variants. As the method is pairwise, we can directly estimate the transmission fitness of the adjacent variants such as DPGR_Alpha,Beta_, DPGR_Beta, Delta_ and DPGR_Delta.Omicron_. To infer the transmission fitness of the nonadjacent variants, DPGR_Alpha,Delta_ and DPGR_Alpha, Omicron_, the property of logarithms (log_10_(*a*/*b*) = log_10_(*a*/*c*) + log_10_(*c*/*b*) is used. Thus, we can infer the DPGR of nonoverlapping variants. This technique allows us to create a distance matrix with a predefined root, capturing the relative fitness of multiple variants over a certain period. For our case, we considered Alpha, the earliest variant, as the root variant (Table 1). The distance matrix is nonnegative because we considered absolute pairwise fitness.

**TABLE 1:** Pairwise distance matrix with the Alpha variant as the root variant.

Variant	Alpha	Beta	Delta	Omicron
Alpha	0.000000	0.002313	0.008400	0.017715
Beta	0.002313	0.000000	0.006088	0.015403
Delta	0.008400	0.006088	0.000000	0.009315
Omicron	0.017715	0.015403	0.009315	0.000000

### 5.6 Construction of fitness stair

The fitness stair (Figure 5D) is a step plot generated using the plt.step() function from the Matplotlib library. This plot visually represents the progressive fitness growth of various COVID‐19 variants of concern (VOCs) relative to a root variant, in this case, Alpha.

By examining the absolute pairwise distance matrix (Table 1), the fitness values for subsequent variants—Beta, Delta, and Omicron—progressively increase relative to the Alpha variant. Specifically, the pairwise distances are 0.002313 for the Beta, 0.008400 for the Delta, and 0.017715 for the Omicron variants. The distance between the Alpha variant and itself is 0 as it serves as the reference point. This fitness stair (Figure 5D) effectively illustrates how each variant diverges in terms of fitness from the original Alpha variant, providing a clear visual representation of the evolutionary changes in their relative fitness.

### 5.7 Construction of fitness landscape

In evolutionary biology, reproductive success of the organisms is understood by the fitness landscape. In the fitness landscape, the height of the landscape defines the relative fitness of one genotype with respect to the others. In our study, using the pairwise absolute distance matrix (Table 1), we constructed a pairwise fitness landscape to understand the relative fitness of pairs of variants. The variant pairs on the top of the landscape have higher relative fitness than those on the bottom of the landscape.

The fitness landscape is generated by reading the distance matrix (Table 1) from a CSV file. This distance matrix, that contains pairwise fitness values between different SARS‐CoV‐2 variants, is used to create a 3D surface plot. The Python pandas library is utilized to load the data and set the appropriate columns as the DataFrame’s index. The *x* and *y* coordinates for the plot are derived from the matrix’s columns and index, respectively. Using Plotly’s ‘go.Surface’ function, a surface trace is created which visualizes the fitness values as a continuous three dimensional surface. The plot is configured to have transparent axes and a background, ensuring a clean visualization focused on the fitness landscape itself. The camera position and perspective are adjusted to provide an optimal view of the surface to understand the relative fitness growth of the pairs of variants. To create the fitness landscape, we used the absolute pairwise distance matrix to visualize the landscape. As we go toward the peaks we observe the pairs with higher relative fitness. For instance, in Figure 5D, Omicron versus Alpha has higher relative fitness than Delta versus Omicron. This comprehensive visualization effectively highlights the relative fitness of each variant in a three‐dimensional space, offering valuable insights into their evolutionary dynamics.

## AUTHOR CONTRIBUTIONS

**Md Jubair Pantho**: Investigation; methodology; software; validation; writing — original draft. **Richard Annan**: Investigation; software; writing — review and editing. **Landen Alexander Bauder**: Investigation; software. **Sophia Huang**: Investigation; visualization. **Letu Qingge**: Funding acquisition; project administration; supervision; validation; writing — review and editing. **Hong Qin**: Conceptualization; formal analysis; funding acquisition; investigation; methodology; project administration; software; supervision; validation; writing — original draft; writing — review and editing.

## CONFLICT OF INTEREST STATEMENT

The authors declare no conflicts of interest.

## ETHICS STATEMENT

This article does not contain any studies with human or animal materials performed by any of the authors.

## Supporting information

Supporting Information S1

## Data Availability

Sample code and key estimates are provided at GitHub (QinLab/DPGR2024). The GISAID data can be accessed at the GISAID website.
