# Supplementary material for: A data‐driven sliding‐window pairwise comparative approach for the estimation of transmission fitness of SARS‐CoV‐2 variants and construction of the evolution fitness landscape
Source: Quant Biol. 2025 Apr 21;13(4):e70003. doi: 10.1002/qub2.70003 (PMC12806054; doi:10.1002/qub2.70003)
Supplement: Supplementary file 1 — Supporting Information S1 [file QUB2-13-e70003-s001.pdf]

Supporting Document

A Data-Driven Sliding-window Pairwise Comparative Approach for the Estimation of Transmission Fitness of SARS-CoV-2 Variants and Construction of the Evolution Fitness Landscape

Md Jubair Pantho, Richard Annan, Landen Alexander Bauder, Sophia Huang, Letu Qingge, Hong Qin

Table of Contents

1 Construction of the Differential Population Growth Rate (DPGR) Model **2**

2. Indirect Estimation of DPGR **3**

3 Mitigating the Sampling Biases in Genomic Surveillance Dataset and Validating Model Robustness against Noise **4**

4. Supporting Figures **5**

Figure S1: Pairwise Transmission fitness estimation of Omicron compared to Delta in several countries **5**

Figure S2 Heatmaps of the estimated pairwise transmission fitness of the WHO labeled of Concern (Variants VOCs) for the target countries **6**

Figure S3: Pairwise Transmission fitness estimation of GRA compared to GK in target countries **7**

Figure S4: Heatmaps of the estimated pairwise transmission fitness of the GISAID labeled clades containing Variants of Concern (VOCs) for the target countries. **8**

Figure S5: Pairwise Transmission fitness estimation of GRA compared to GK in target continents **9**

Figure S6: Heatmaps of the estimated pairwise transmission fitness of the GISAID labeled clades containing Variants of Concern (VOCs) for the target continents. **10**

Figure S7: Pairwise Transmission fitness estimation of Omicron compared to Delta after introducing Gaussian noise in target countries **12**

Figure S8: Estimated pairwise transmission fitness of Delta compared to Omicron in several countries **13**

Figure S9: Estimated pairwise transmission fitness of Delta compared to Omicron in several continents **14**

Figure S10: Estimated pairwise transmission fitness of GK Compared to GRA in several countries **15**

Figure S11 : Fitness stair of Omicron Sub-lineages (BA.1*, BA.2*, BA.4*, BA.5*) 16
Figure S12: Estimated transmission fitness comparison between DPGR and PyRO model . 17
Figure S13: Estimated pairwise transmission fitness of GK Compared to GRA in several continents 18
Figure S14: Sensitivity test of sliding windows shows broad ranges to satisfy the log-linearity assumption of DPGR... 19
Figure S15: Faster DPGR can dominate smaller ones in simulated data sets. 20
5. Supporting Tables 21
Table S1: Fitting results for Omicron vs. Delta variations at different locations.......... 21
Table S2: Fitting results for Omicron sub-lineages (BA.1* -BA.5*) at different geographic locations 22
Table S3: Influence of moderate Gaussian noise on DPGR estimation 23
Table S4: Fitting results for GRA Vs. GK at Different Geographic Locations 24
Table S5: DPGR Estimates for All Variant Pairs (WHO Label)...................................... 25

1 Construction of the Differential Population Growth Rate (DPGR) Model

When estimating the Differential Population Growth Rate (DPGR) between two target variants, it is assumed that the populations exhibit exponential growth rates. A period of linear growth is selected for analysis within the time window between pairs of target variants. Additionally, a time lag is accounted for to address the difference in the timing of their growth phases.

$$N_1 = N_{1,0}e^{g_1(t-T_1)}\ , \quad N_2 = N_{2,0}e^{g_2(t-T_2)}$$

where, the variable t is time, N_1 and N_2 are the population as a function of time, $N_{1,0}$ and $N_{2,0}$ are the initial populations, g1 and g2 are the growth rates, and T_1 and T_2 are the time lag of their growth, where $t > T_1$ and T_2.

Now taking the ratio of their growth rate,

$$\frac{N_1}{N_2} = \frac{N_{1,0}}{N_{2,0}} e^{g_1(t-T_1)-g_2(t-T_2)}$$

$$= C_1\ e^{(g_1-g_2)t\, +\, (-g_1T_1+g_2T_2)}$$

$$= C_1 e^{(g_1-g_2)t\, +\, C_2}$$

Here, $C_1 = N_{1,0}/N_{2,0}$ and $C_2 = (-g_1T_1 + g_2T_2)$ are constants. Now taking the log transformation on both sides, we get,

$$log\ log\ \left(\frac{N_1}{N_2}\right) = (g_1 - g_2)t + C \qquad \text{Eq. S1}$$

where C is a constant derived from C_1 and C_2. Because of the asymptotic cases of SAS-CoV-2, it is likely N_1 and N_2 are under-reported, which can lead to sampling biases. If we assume that the sampling biases are invariant or similar among different variants, then the ratio of N_1/N_2 would approximate the true ratio of the two subpopulations of variations.

The term $(g_1 - g_2)$ denotes the Differential Population between the two populations of interest. If g1 is greater than g2, the first population grows faster than the second, and DPGR is a positive value. Otherwise, DPGR would be a negative value.

With the linear form of Eq S1, we can apply linear regression to a scatter plot of log(N1/N2) versus t in an appropriate time window to infer DPGR.

2. Indirect Estimation of DPGR

During the COVID-19 pandemic period, some variants, say N_1, dominated in earlier stages, and some variants, say N_3, appeared much later. Given the limited genomic sampling capacity, we cannot observe N_1/N_3 directly. In this case, we can choose an intermediate variant N_2 through which we can observe N_1/N_2 and N_2/N_3 through genomic sampling. For instance, for SARS-CoV-2 variants, there is limited co-sampling of Alpha and Delta variants in the same location which makes $DPGR_{Alpha\text{-}Delta}$ challenging to estimate. With the Beta variant as an intermediate, we can have $DPGR_{Alpha\text{-}Delta} = DPGR_{Alpha\text{-}Beta} + DPGR_{Beta\text{-}Delta}$.

The inference is based on the property of logarithms indicates, log(a/c) =log(a/b)+log(b/c), and is illustrated as follows.

$$
\begin{aligned}
DPGR_{1\text{-}3} \cdot t + C_{1\text{-}3} &= (g_1 - g_3) \cdot t + (-g_1 T_1 + g_3 T_3) \\
&= \log(N_1/N_3) \\
&= \log((N_1/N_2) \cdot (N_2/N_3)) \\
&= \log(N_1/N_2) + \log(N_2/N_3) \\
&= (g_1 - g_2) \cdot t + (-g_1T_1 + g_2T_2) + (g_2 - g_3) \cdot t + (-g_2T_2 + g_3T_3)) \\
&= DPGR_{1\text{-}2} \cdot t + DPGR_{2\text{-}3} \cdot t + (-g_1T_1 + g_3 T_3)
\end{aligned}
$$

Simplifying, we have,

$$DPGR_{1\text{-}3} \cdot t = (DPGR_{1\text{-}2} + DPGR_{2\text{-}3}) \cdot t$$

Hence,

$$DPGR_{1\text{-}3} = DPGR_{1\text{-}2} + DPGR_{2\text{-}3}$$

3 Mitigating the Sampling Biases in Genomic Surveillance Dataset and Validating Model Robustness against Noise

Estimating the viral transmission fitness is prone to sampling biases. Inconsistency in the data and geographic dominance of one variant over the other can highly compromise the estimated fitness values at a certain region. Moreover, underreporting of the genomic sequencing data is frequently observed. For estimating the pairwise relative transmission fitness - DPGR, the weekly submission counts for any two target variants at each location within a particular time window are considered. This approach essentially uses one variant as an internal reference, which can reduce the biases induced by sampling.

First, to test the model's performance in response to the noise in the data, the preprocessed GISAID dataset is introduced with random noise from the Gaussian distribution. The noise is added to the '*Freq'* column of the dataset, which records the weekly sum of submission counts for each variant observed at a particular location. Gaussian distribution of mean 0 and varying standard deviation depending on the spread of the frequency count in a particular location is selected to induce the noise. The "np.normal.random()" function from the NumPy library is used to select random values from the Gaussian Distribution and added to the existing values of the *Freq* column. After that, the model is fitted with the noisy data to observe the linear performance of the model, as according to the assumption of the DPGR model, the transmission fitness growth must follow a linear pattern. Figures S7 illustrates the transmission fitness estimation scatterplots of Omicron compared to Delta in several countries and continents. It can be observed that after introducing the Gaussian noise, the estimated DPGR value remains in similar ranges and the linearity of the assumption also holds based on R-squared value and p-value (table S3).

4. Supporting Figures

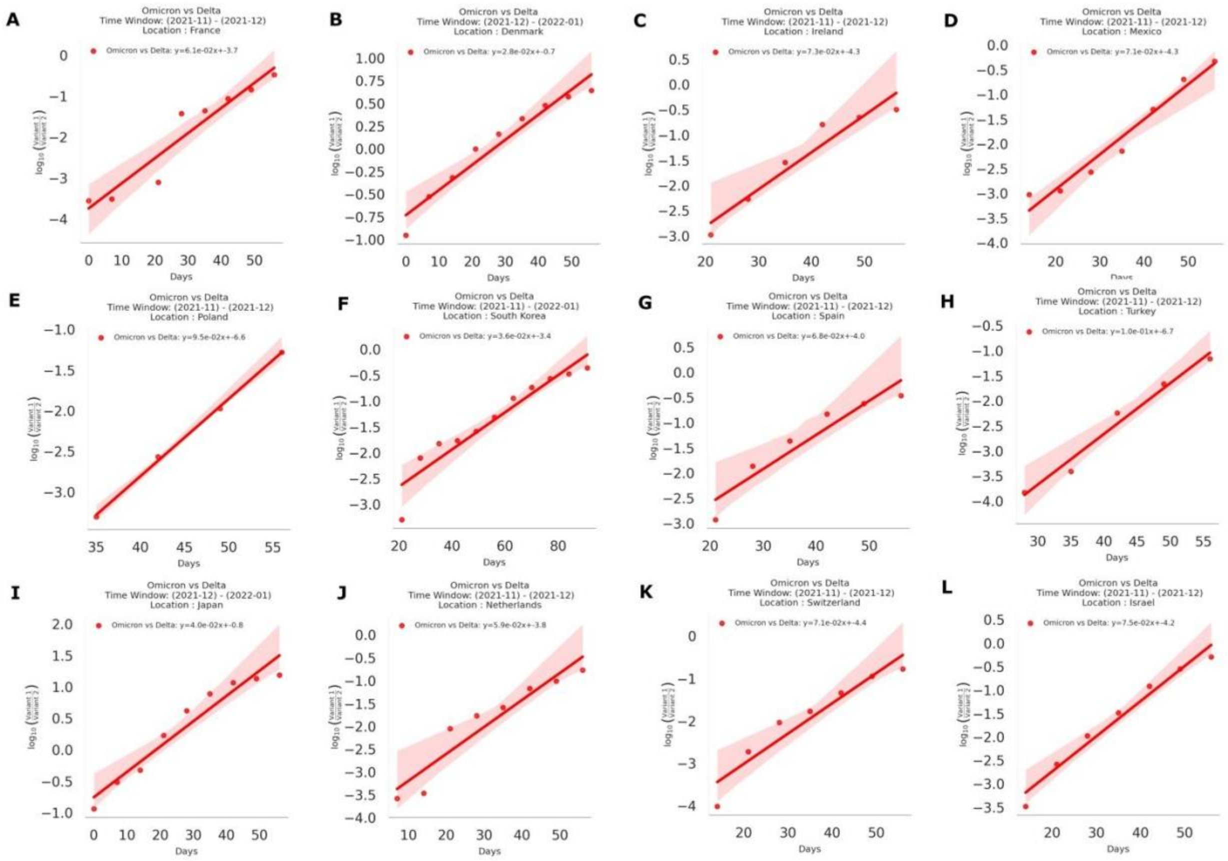

Figure S1: Pairwise Transmission fitness estimation of Omicron compared to Delta in several countries

The subplots (A-L) illustrate the sharp increase of pairwise transmission fitness of Omicron Compared to Delta in the target countries.

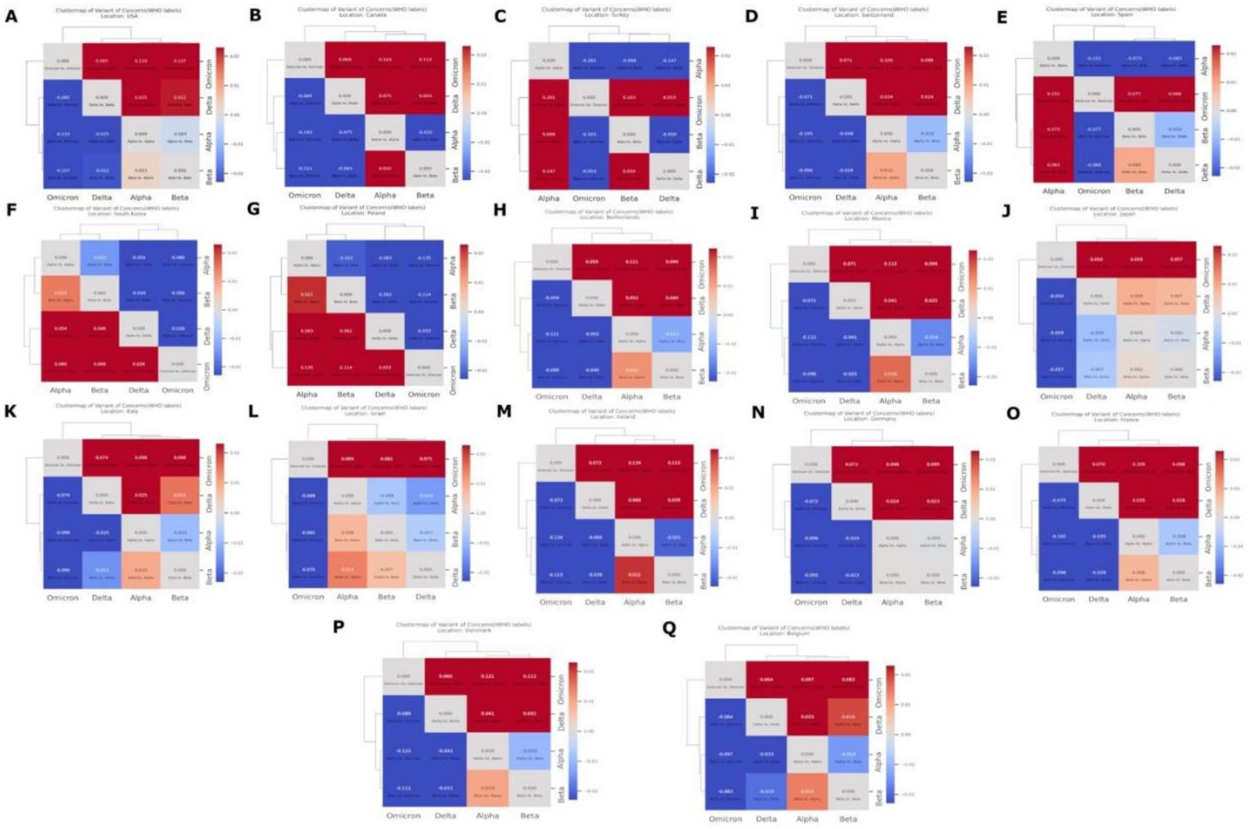

Figure S2 Heatmaps of the estimated pairwise transmission fitness of the WHO labeled of Concern (Variants VOCs) for the target countries.

The heatmaps (A-Q) are read from row to column, describing the transmission fitness of a row variant compared to a variant in a column. The dark red cells in the figures indicate higher transmission fitness of the specific variant in the row compared to the variant in the column. Cells colored in blue indicate that a particular variant in the row has negative transmission fitness growth with the variant in the column. Dendrograms are plotted on the left and the top of each figure to depict differences in the transmission fitness growth rate of one variant to another. A higher variant distance indicates a significant difference in growth. Variants under the same node are closer to each other in transmission fitness growth than the other variants.

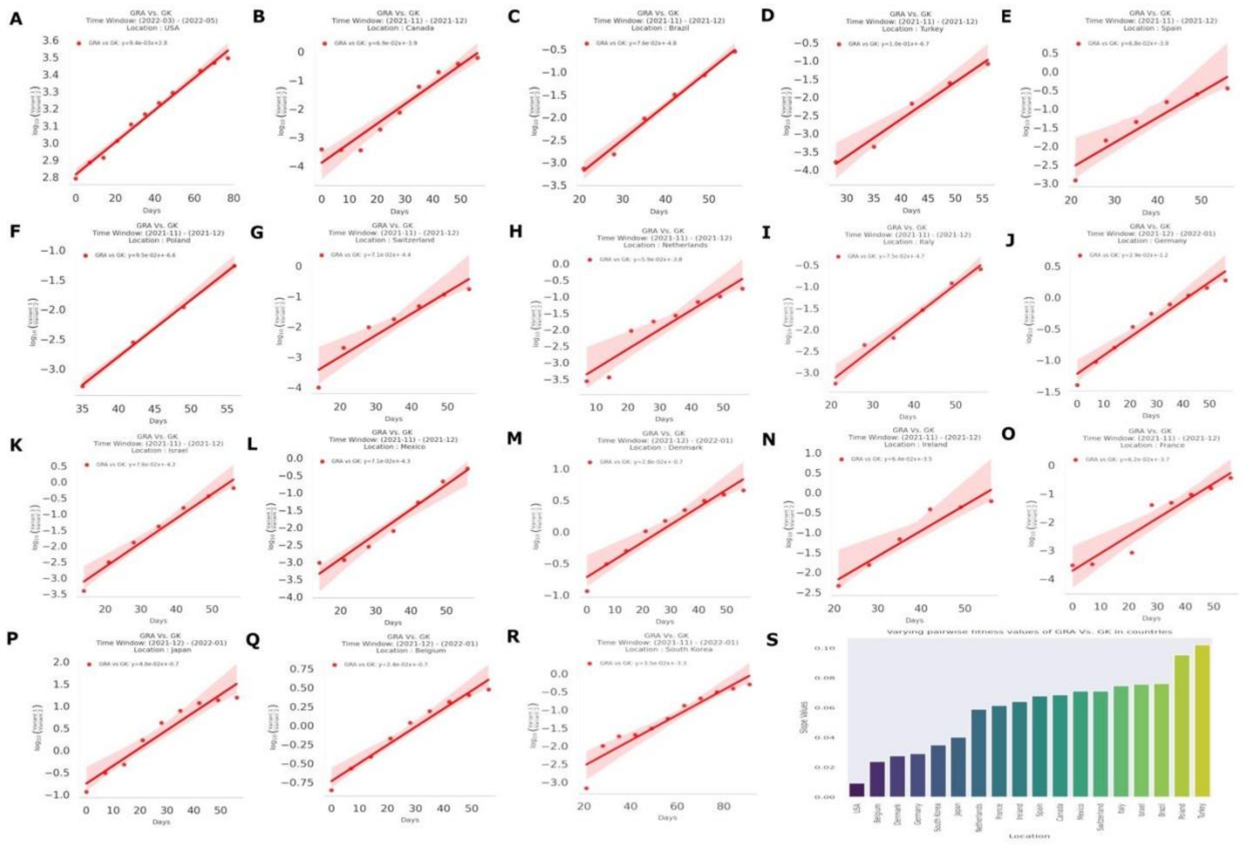

Figure S3: Pairwise Transmission fitness estimation of GRA compared to GK in target countries

The sub-plots **(A-G)** illustrate the estimated pairwise transmission fitness of GRA Compared to GK in the target countries. G. The bar plot visualizes the estimated transmission fitness values in all the analyzed regions.

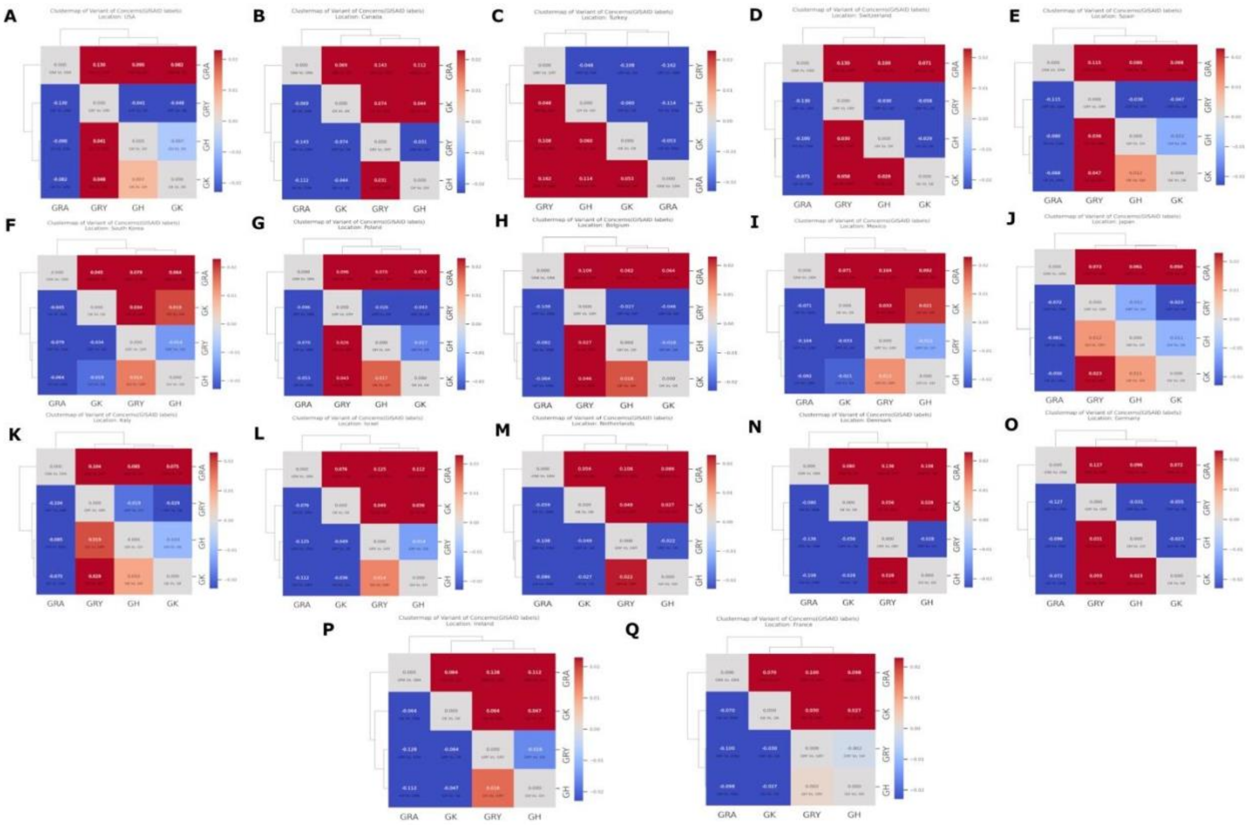

Figure S4: Heatmaps of the estimated pairwise transmission fitness of the GISAID labeled clades containing Variants of Concern (VOCs) for the target countries.

The heatmaps from **(A-Q)** are read from row to column, describing the transmission fitness of a row variant compared to a variant in a column. The dark red cells in the figures indicate higher transmission fitness of the specific clade in the row compared to the clade in the column. Cells colored in blue indicate that a particular clade in the row has negative transmission fitness growth with the corresponding clade in the column. Dendrograms are plotted on the left and the top of each figure to depict differences in the transmission fitness growth rate of one clade to another. A higher clade distance indicates a significant difference in transmission fitness growth. Clades under the same node are closer to each other in transmission fitness growth than the other clades.

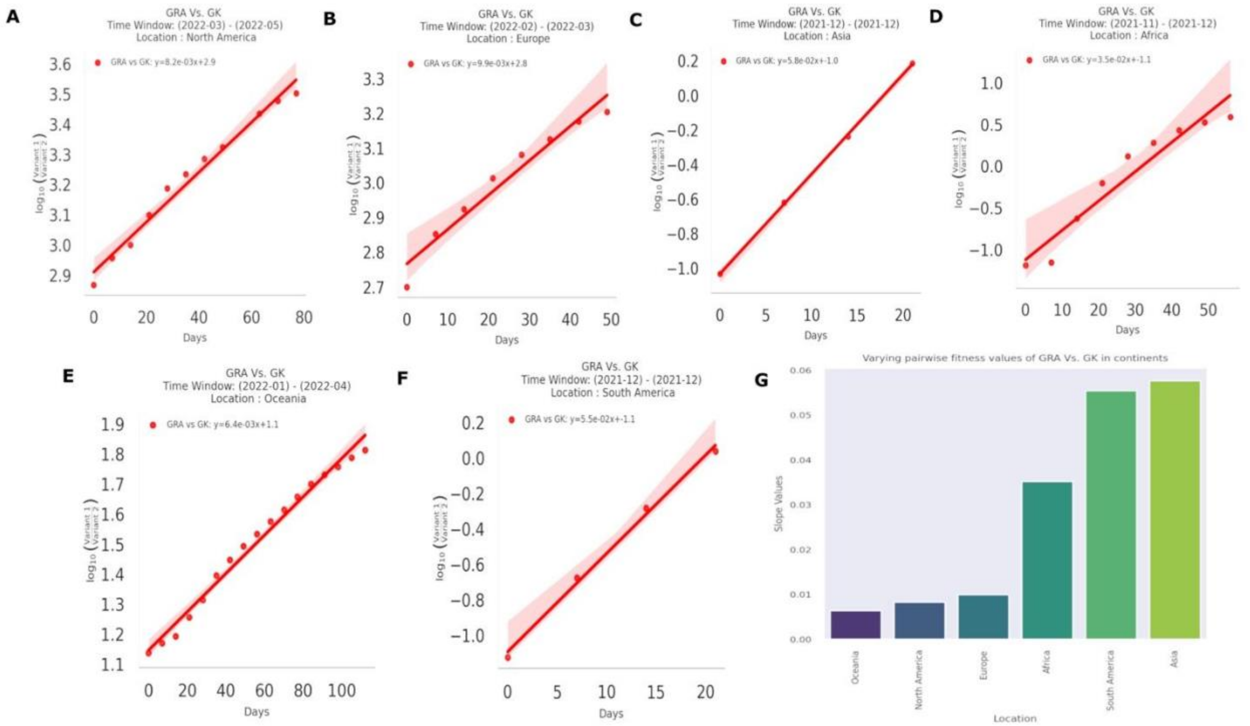

Figure S5: Pairwise Transmission fitness estimation of GRA compared to GK in target continents

The sub-plots **(A-F)** illustrate the sharp increase of pairwise transmission fitness of GRA Compared to GK in the target continents. **G.** The bar plot visualizes the estimated transmission fitness values in all the analyzed regions

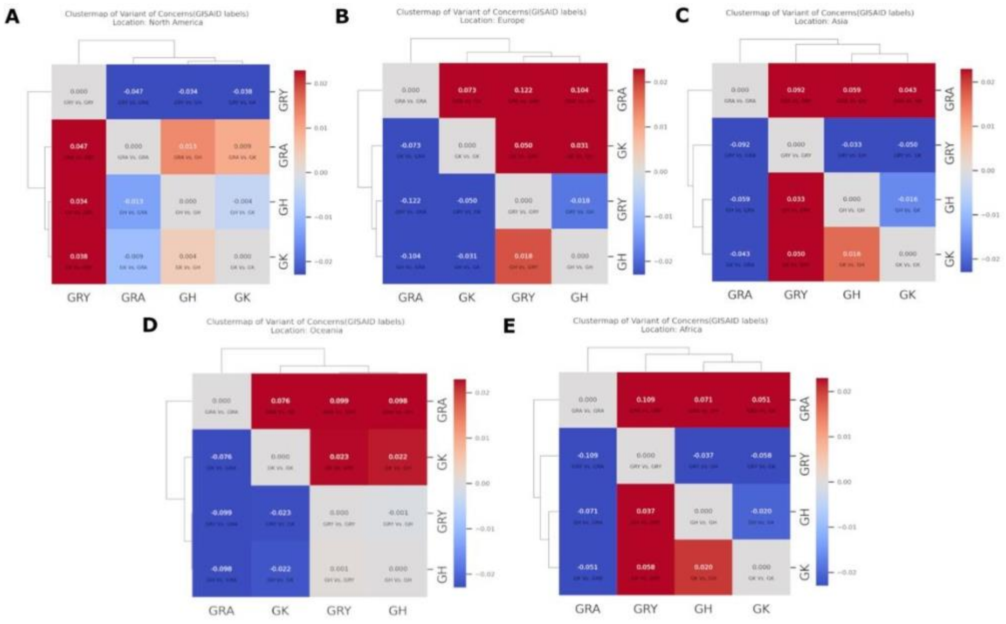

Figure S6: Heatmaps of the estimated pairwise transmission fitness of the GISAID labeled clades containing Variants of Concern (VOCs) for the target continents.

The heatmaps from **(A-E)** are read from row to column, describing the transmission fitness of a row clade compared to a clade in a column. The dark red cells in the figures indicate higher transmission fitness of the specific clade in the row compared to the clade in the column. Cells colored in blue indicate that a particular clade in the row has negative transmission fitness growth with the corresponding clade in the column. Dendrograms are plotted on the left and the top of each figure to depict differences in the transmission fitness growth rate of one clade to another. A higher clade distance indicates a significant difference in transmission fitness growth. Clades under the same node are closer to each other in transmission fitness growth than the other clades.

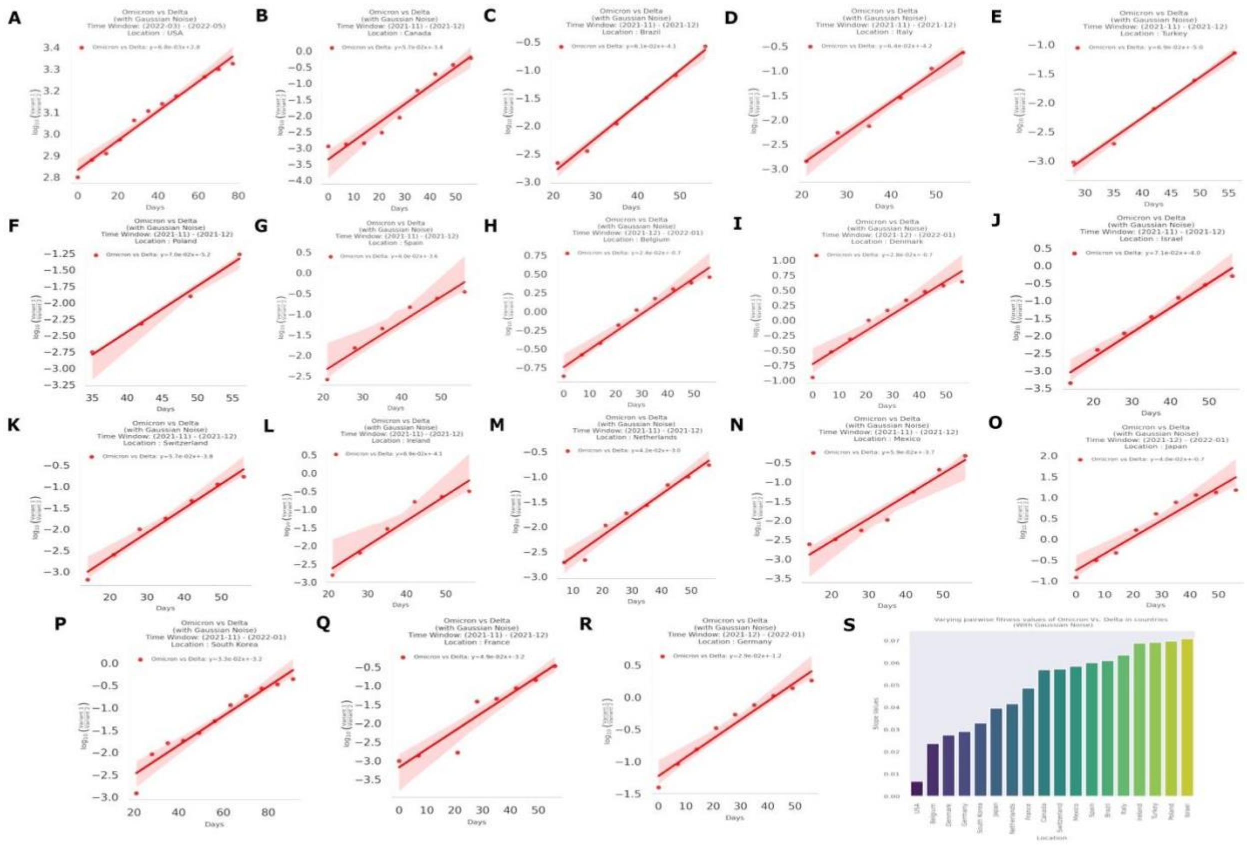

Figure S7 : Pairwise Transmission fitness estimation of Omicron compared to Delta after introducing Gaussian noise in target countries

The estimated transmission fitness plots **(A-F)** of Omicron Compared to Delta in the target different continents after introducing Gaussian noise. **G**. Bar plot visualizes the estimated transmission fitness values in all the analyzed regions.

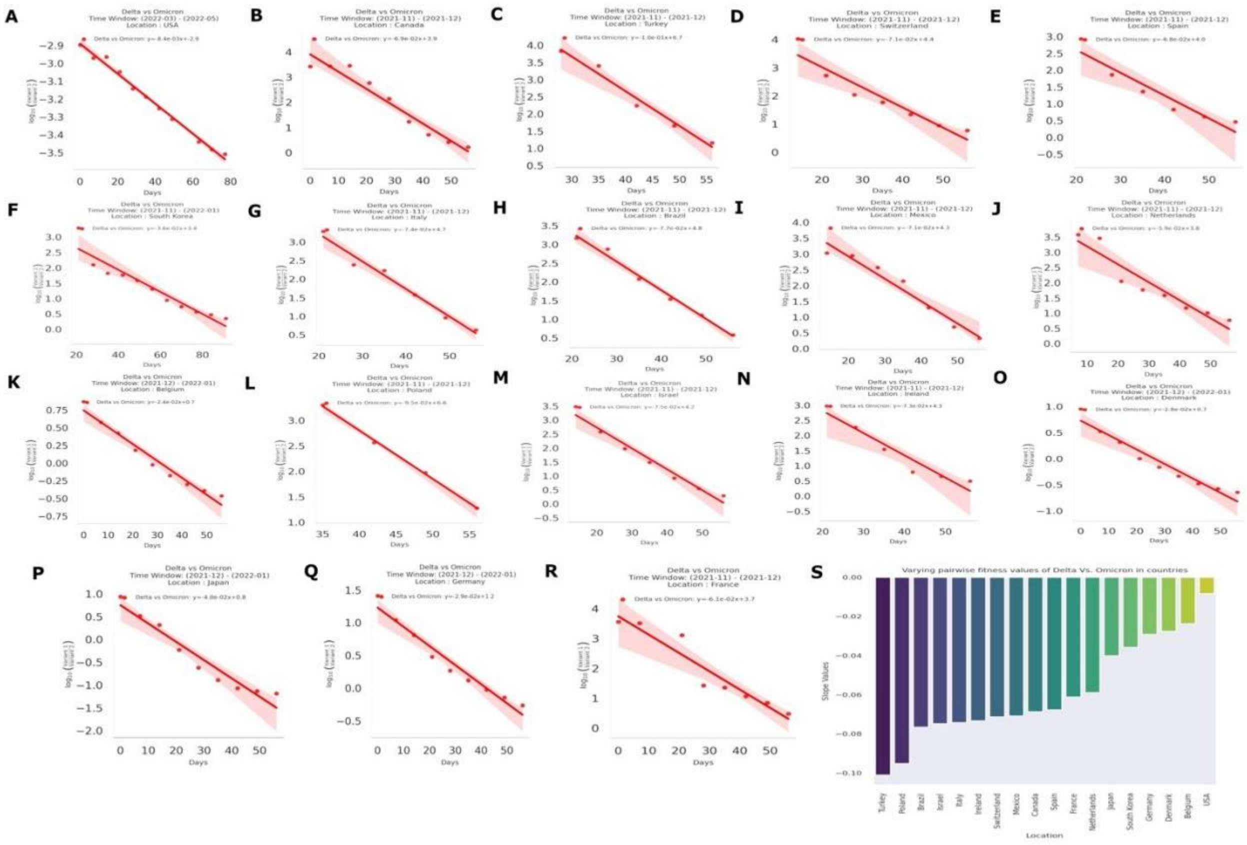

Figure S8: Estimated pairwise transmission fitness of Delta compared to Omicron in several countries

The sub-plots **(A-R)** illustrate transmission fitness plots of Delta Compared to Omicron. The negative trend line indicates Delta has a negative transmission fitness growth compared to Omicron in different time windows in the selected regions. **G.** Bar plot visualizes the estimated transmission fitness values in all the analyzed regions.

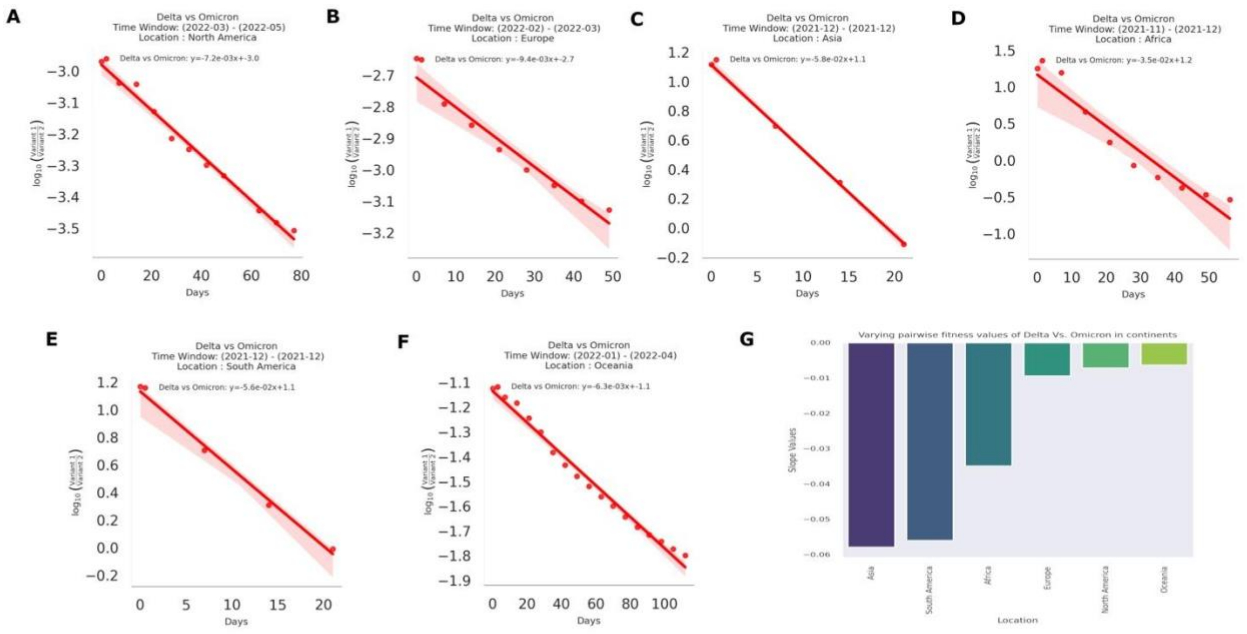

Figure S9: Estimated pairwise transmission fitness of Delta compared to Omicron in several continents

The sub-plots (A-G) illustrate transmission fitness plots of Delta Compared to Omicron in the target continents. The negative trend line indicates Delta has a negative transmission fitness growth compared to Omicron in different time windows in the selected regions. G. Bar plot visualizes the estimated transmission fitness values in all the analyzed regions.

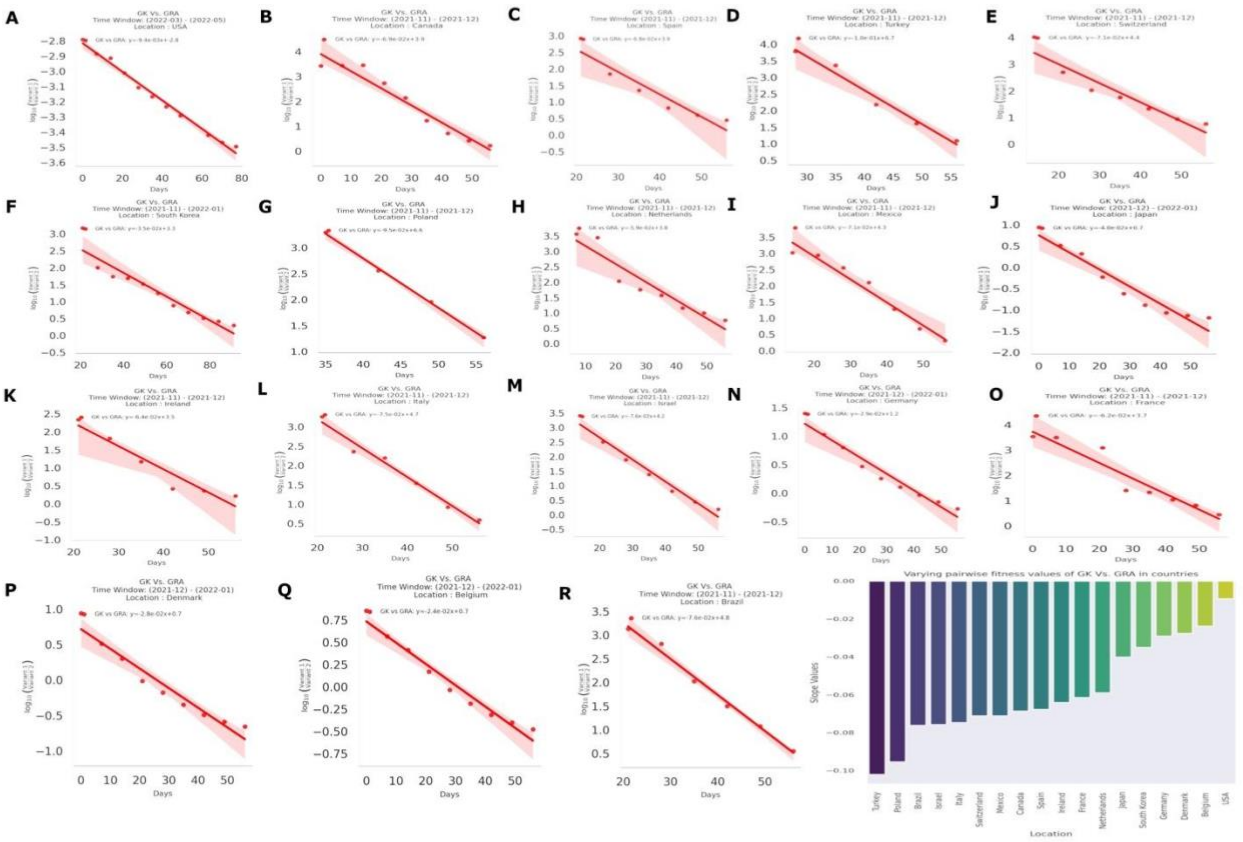

Figure S10: Estimated pairwise transmission fitness of GK Compared to GRA in several countries

The sub-plots **(A-R)** illustrate transmission fitness plots of GISAID clades GK Compared to GRA in the target countries. The negative trend line indicates GK has a negative transmission fitness growth compared to GRA in different time windows in the selected regions. **The** Bar plot visualizes the estimated transmission fitness values in all the analyzed regions.

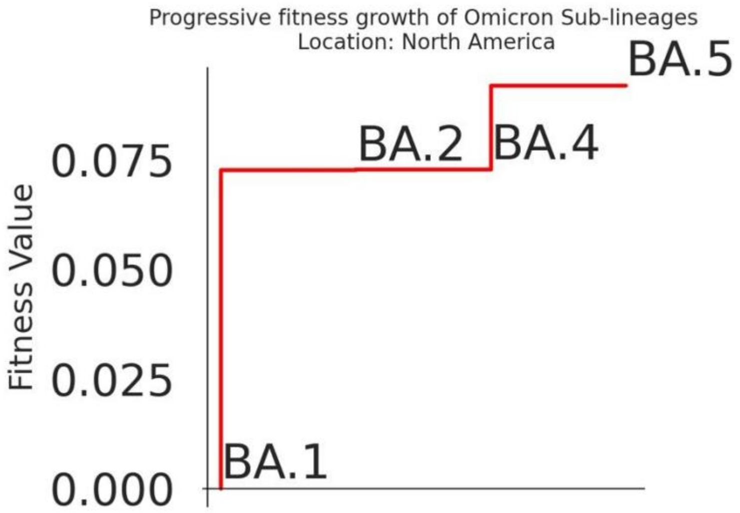

Figure S11 : Fitness stair of Omicron Sub-lineages (BA.1*, BA.2*, BA.4*, BA.5*)

The fitness stair illustrates the progressive transmission fitness gain of the Sub-lineages of the Omicron variant in the United States.

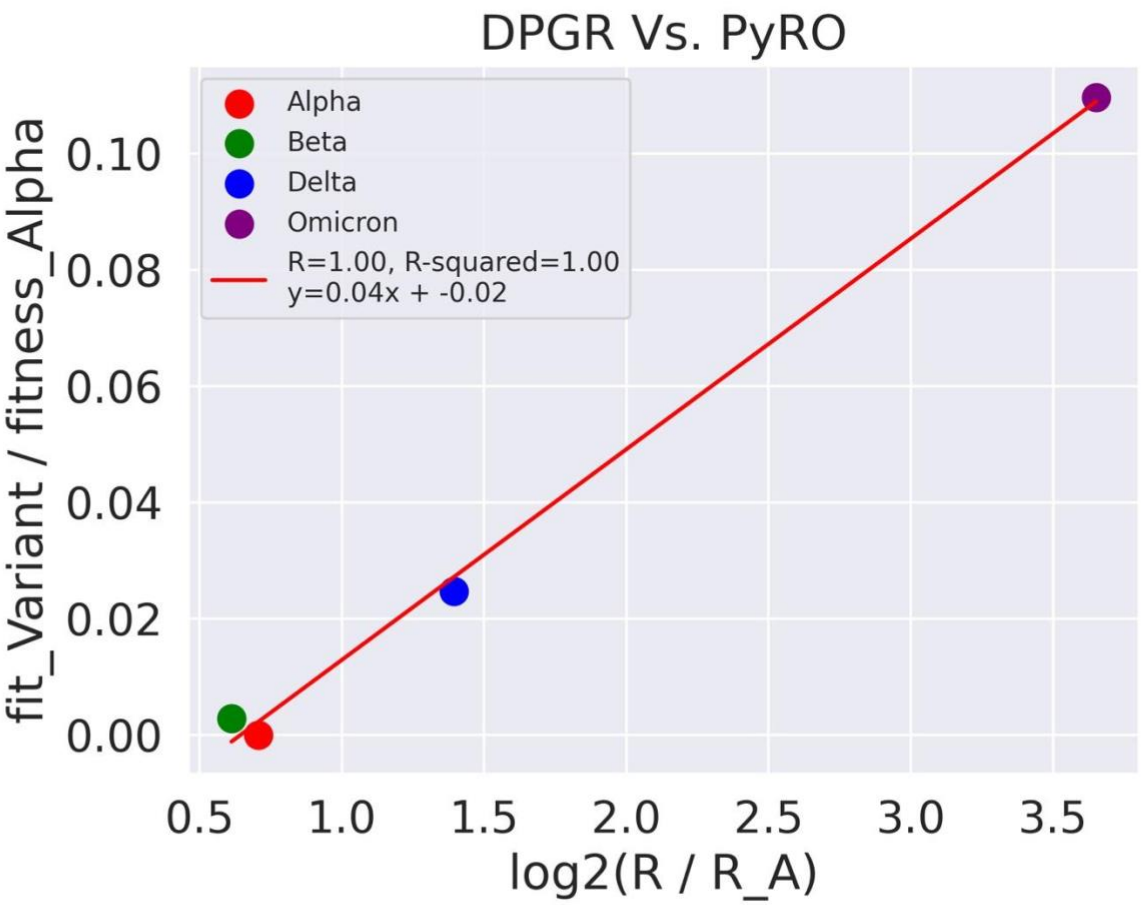

Figure S12: Estimated transmission fitness comparison between DPGR and PyRO model

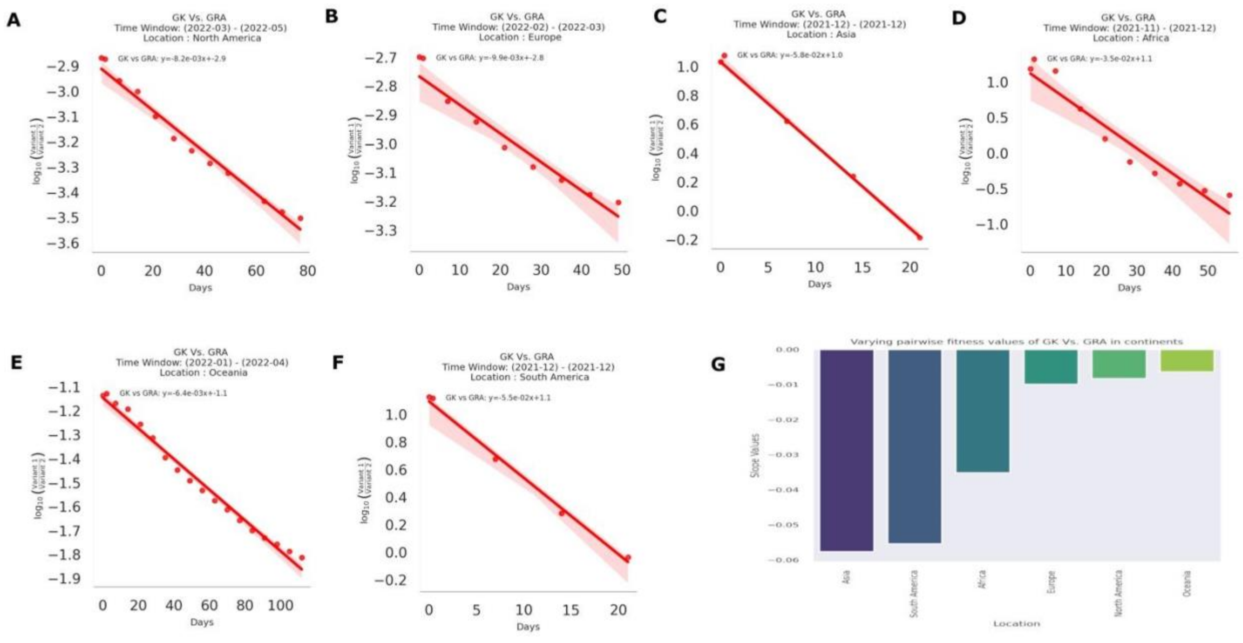

Figure S13: Estimated pairwise transmission fitness of GK Compared to GRA in several continents

The sub-plots **(A-F)** illustrate transmission fitness plots of GISAID clades GK Compared to GRA in the target continents. The negative trend line indicates GK has a negative transmission fitness growth compared to GRA in different time windows in the selected regions. **G.** Bar plot visualizes the estimated transmission fitness values in all the analyzed regions.

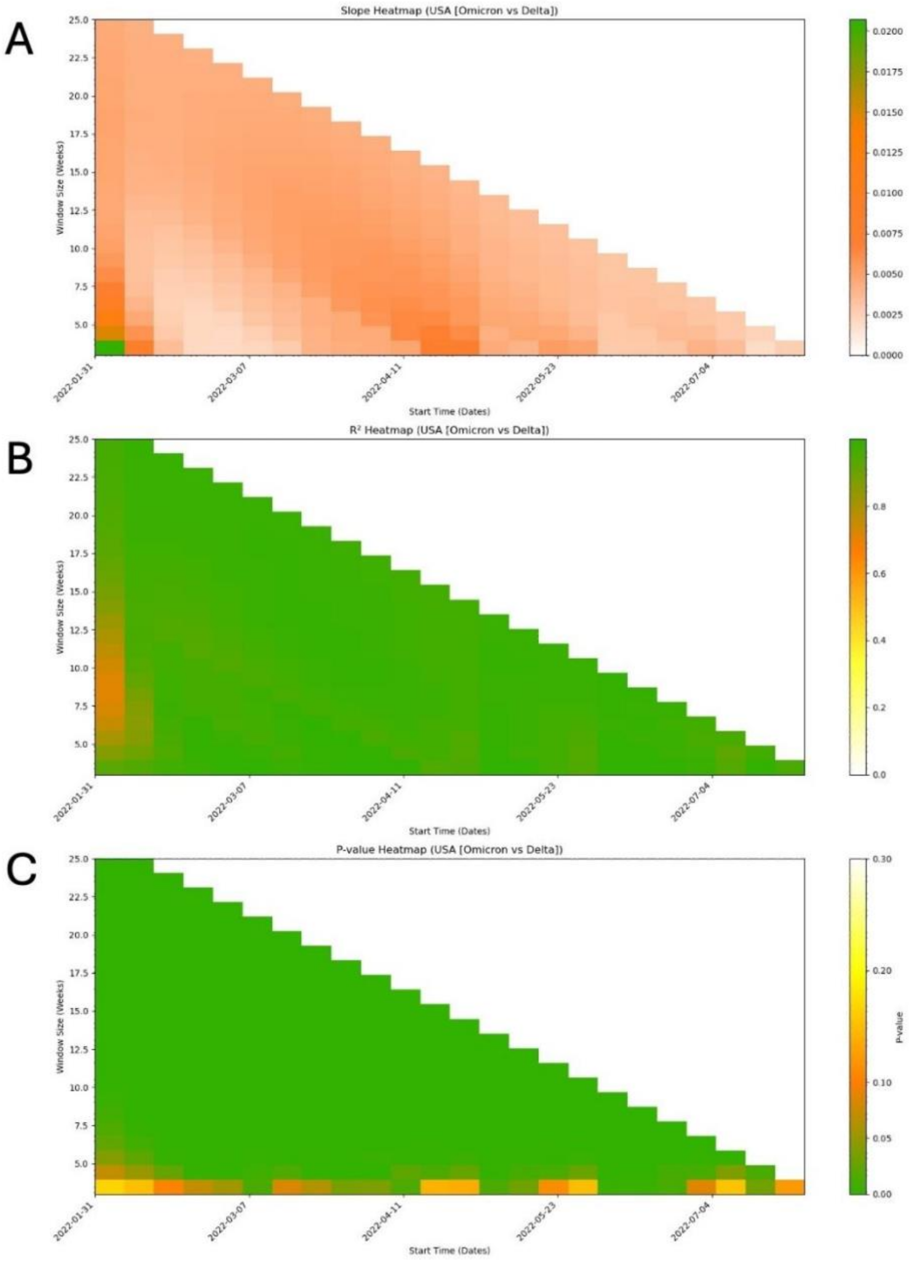

Figure S14: Sensitivity test of sliding windows shows broad ranges to satisfythe log-linearity assumption of DPGR.

Using the Omicron-Delta variants in the USA as an example, we found that log-linearity assumption for DPGR can hold in a broad range of parameter combinations of starting window time and length of windows based on (A) Slopes, (B) R^2 values, and (C) the p-values of the DPGR log-linear fit. Overall, we can see sliding windows smaller than 5 often lead to a p-value greater than 0.05 (C). Overall, the slope ranges with a sliding window size greater than 5 are stable around 0.008.

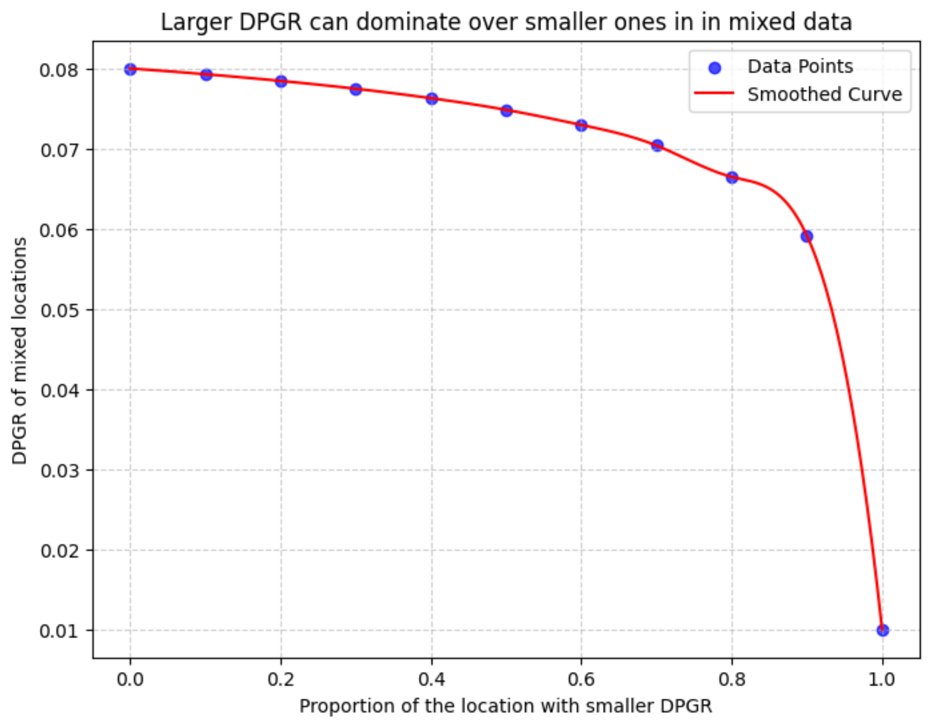

Figure S15: Faster DPGR can dominate smaller ones in simulated data sets.

Two locations were simulated with DPGR at 0.08 and 0.01 for variants 1 and 2. The simulation then examined various proportions of mixing the two locations, ranging from 0%-100%, 10% - 90%, 20% - 80%, …, to 90%-0%, and 100% - 0%. It can be seen that the estimated DPGR from the mixture was in the range of 0.6 ~ 0.8 in most of the combinations, demonstrating the faster DPGR typically overshadows the population with a smaller DPGR.

5. Supporting Tables

Table S1: Fitting results for Omicron vs. Delta variations at different locations.

Location (Country/Continents)	P-Value	Slope Value	R^2 value	Time Window
USA	0.0000000002	0.0084	0.99	'2022-03' - '2022-05'
Canada	0.0000121512	0.0688	0.94	'2021-11' - '2021-12'
Brazil	0.0000424733	0.0767	0.98	'2021-11' - '2021-12'
Germany	0.0000037926	0.0292	0.96	'2021-12' - '2022-01'
Belgium	0.0000017600	0.0239	0.97	'2021-12' - '2022-01'
Italy	0.0002011518	0.0743	0.98	'2021-11' - '2021-12'
Turkey	0.0017071813	0.1011	0.97	'2021-11' - '2021-12'
Israel	0.0000498947	0.0750	0.97	'2021-11' - '2021-12'
Ireland	0.0019830378	0.0734	0.93	'2021-11' - '2021-12'
Spain	0.0034588120	0.0680	0.91	'2021-11' - '2021-12'
France	0.0002099997	0.0612	0.91	'2021-11' - '2021-12'
Denmark	0.0000116662	0.0280	0.94	'2021-12' - '2022-01'
South Korea	0.0000074797	0.0360	0.90	'2021-11' - '2022-01'
Japan	0.0000169371	0.0402	0.94	'2021-12' - '2022-01'
Netherlands	0.0003426374	0.0590	0.898	'2021-11' - '2021-12'
Switzerland	0.0008614151	0.0714	0.91	'2021-11' - '2021-12'
Poland	0.0007941103	0.0952	0.99	'2021-11' - '2021-12'
Mexico	0.0001007758	0.0709	0.96	'2021-11' - '2021-12'
North America	0.0000000006	0.0072	0.99	'2022-03' - '2022-05'
South America	0.0032756997	0.0561	0.99	'2021-12'
Europe	0.0000234708	0.0094	0.96	'2022-02' - '2022-03'
Oceania	0.0000000000	0.0063	0.99	'2022-01', '2022-02', '2022-03', '2022-04'
Asia	0.0001804061	0.0579	0.98	'2021-12'
Africa	0.0000349938	0.0349	0.92	'2021-11' - '2021-12'

Table S2: Fitting results for Omicron sub-lineages (BA.1* -BA.5*) at different geographic locations

Location (Continent)	Comparison	P-Value	Slope Value	R^2 value	Time Window
North America	BA.5 vs BA.1	0.0000017075	0.053027	0.982	'2022-04' – '2022-05'
North America	BA.5 vs BA.2	0.0000008811	0.042768	0.985	'2022-04' – '2022-05'
North America	BA.5 vs BA.3	0.0002200994	0.052902	0.993	'2022-04' – '2022-05'
North America	BA.5 vs BA.4	0.0001053811	0.013199	0.931	'2022-04' – '2022-05'
Europe	BA.5 vs BA.1	0.0000000001	0.045530	0.986	'2022-02' – '2022-05'
Europe	BA.5 vs BA.2	0.0000000001	0.042186	0.988	'2022-02' – '2022-05'
Europe	BA.5 vs BA.3	0.0000000019	0. 047117	0.990	'2022-02' – '2022-05'
Europe	BA.5 vs BA.4	0.0047140545	0. 003869	0.567	'2022-02' – '2022-05'
Asia	BA.5 vs BA.1	0.0000056861	0.057299	0.974	'2022-03' – '2022-05'
Asia	BA.5 vs BA.2	0.0000041944	0.046805	0.959	'2022-03' – '2022-05'
Asia	BA.5 vs BA.3	0.0024389686	0.051755	0.968	'2022-03' – '2022-05'
Asia	BA.5 vs BA.4	0.2765494733	0.005945	0.193	'2022-03' – '2022-05'
Africa	BA.5 vs BA.1	0.0000000005	0.033623	0.988	'2022-02' – '2022-04'
Africa	BA.5 vs BA.2	0.0000000042	0.027082	0.981	'2022-02' – '2022-04'
Africa	BA.5 vs BA.3	0.0002743433	0.033385	0.993	'2022-02' – '2022-04'
Oceania	BA.5 vs BA.1	0.0000000300	0.048157	0.995	'2022-03' – '2022-05'
Oceania	BA.5 vs BA.2	0.0000000255	0.043159	0.995	'2022-03' – '2022-05'

Table S3: Influence of moderate Gaussian noise on DPGR estimation

Location (Country)	Comparison	P-Value	Slope Value	R^2 value	Time Window
USA	Omicron Vs. Delta	0.0000000067	0.008	0.98	'2022-03' - '2022-05'
Canada	Omicron Vs. Delta	0.0046773058	0.009	0.70	'2021-11' - '2021-12'
Brazil	Omicron Vs. Delta	0.0463248792	0.006	0.67	'2021-11' - '2021-12'
Germany	Omicron Vs. Delta	0.0000215862	0.031	0.93	'2021-12' - '2022-01'
Belgium	Omicron Vs. Delta	0.0000030266	0.051	0.96	'2021-12' - '2022-01'
Italy	Omicron Vs. Delta	0.0268002067	0.006	0.75	'2021-11' - '2021-12'
Turkey	Omicron Vs. Delta	0.0818599314	0.003	0.84	'2021-11' - '2021-12'
Israel	Omicron Vs. Delta	0.0104577546	0.014	0.84	'2021-11' - '2021-12'
Ireland	Omicron Vs. Delta	0.0024074943	0.009	0.92	'2021-11' - '2021-12'
Spain	Omicron Vs. Delta	0.0019413457	0.010	0.93	'2021-11' - '2021-12'
France	Omicron Vs. Delta	0.0146589756	0.005	0.66	'2021-11' - '2021-12'
Denmark	Omicron Vs. Delta	0.0000037399	0.081	0.96	'2021-12' - '2022-01'
South Korea	Omicron Vs. Delta	0.0000567553	0.006	0.85	'2021-11' - '2022-01'
Japan	Omicron Vs. Delta	0.0000151458	0.04	0.94	'2021-12' - '2022-01'
Netherlands	Omicron Vs. Delta	0.0027182864	0.003	0.86	'2021-11' - '2021-12'
Switzerland	Omicron Vs. Delta	0.0055076478	0.004	0.81	'2021-11' - '2021-12'
Poland	Omicron Vs. Delta	0.1311799352	0.002	0.76	'2021-11' - '2021-12'
Mexico	Omicron Vs. Delta	0.0263814437	0.009	0.66	'2021-11' - '2021-12'

Table S4: Fitting results for GRA Vs. GK at Different Geographic Locations

Location (Country/Continents)	P-Value	Slope Value	R^2 value	Time Window
USA	0.0000000003	0.0094	0.99	'2022-03' - '2022-05'
Canada	0.0000110765	0.0687	0.95	'2021-11' - '2021-12'
Brazil	0.0000332042	0.0762	0.99	'2021-11' - '2021-12'
Germany	0.0000038165	0.0292	0.96	'2021-12' - '2022-01'
Belgium	0.0000017206	0.0239	0.97	'2021-12' - '2022-01'
Italy	0.0001901356	0.0747	0.98	'2021-11' - '2021-12'
Turkey	0.0017446336	0.1021	0.97	'2021-11' - '2021-12'
Israel	0.0000478457	0.0758	0.97	'2021-11' - '2021-12'
Ireland	0.0023451291	0.0641	0.92	'2021-11' - '2021-12'
Spain	0.0035146910	0.0678	0.90	'2021-11' - '2021-12'
France	0.0002081598	0.0615	0.91	'2021-11' - '2021-12'
Denmark	0.0000116616	0.0277	0.94	'2021-12' - '2022-01'
South Korea	0.0000069824	0.0351	0.90	'2021-11' - '2022-01'
Japan	0.0000169799	0.0402	0.94	'2021-12' - '2022-01'
Netherlands	0.0003454587	0.0590	0.89	'2021-11' - '2021-12'
Switzerland	0.0009175994	0.0711	0.91	'2021-11' - '2021-12'
Poland	0.0007546938	0.0954	0.99	'2021-11' - '2021-12'
Mexico	0.0000858866	0.0711	0.96	'2021-11' - '2021-12'
North America	0.0000000052	0.0082	0.98	'2022-03' - '2022-05'
South America	0.0028071909	0.0554	0.99	'2021-12'
Europe	0.0000376295	0.0099	0.95	'2022-02' - '2022-03'
Oceania	0.0000000000	0.0064	0.99	'2022-01', '2022-02', '2022-03', '2022-04'
Asia	0.0001752538	0.0577	0.99	'2021-12'
Africa	0.0000328541	0.0351	0.93	'2021-11' - '2021-12'

Table S5: DPGR Estimates for All Variant Pairs (WHO Label)

Location(Continent)	Comparison	Slope Value
North America	Alpha Vs. Alpha	NA
	Beta Vs. Alpha	0.000392
	Delta Vs. Alpha	0.006577
	Omicron Vs. Alpha	0.014587
	Beta Vs. Beta	NA
	Delta Vs. Beta	0.006185
	Omicron Vs. Beta	0.014195
	Delta Vs. Delta	NA
	Omicron Vs. Delta	0.008010
	Omicron Vs. Omicron	NA
Europe	Alpha Vs. Alpha	NA
	Beta Vs. Alpha	0.012519
	Delta Vs. Alpha	0.044362
	Omicron Vs. Alpha	0.117089
	Beta Vs. Beta	A
	Delta Vs. Beta	0.031843
	Omicron Vs. Beta	0.104570
	Delta Vs. Delta	NA
	Omicron Vs. Delta	0.072727
	Omicron Vs. Omicron	0.00
Asia	Alpha Vs. Alpha	NA
	Beta Vs. Alpha	0.004142
	Delta Vs. Alpha	0.012872
	Omicron Vs. Alpha	0.057688
	Beta Vs. Beta	NA
	Delta Vs. Beta	0.008729
	Omicron Vs. Beta	0.053545
	Delta Vs. Delta	NA
	Omicron Vs. Delta	0.044816
	Omicron Vs. Omicron	NA
Africa	Alpha Vs. Alpha	NA
	Beta Vs. Alpha	0.027493
	Delta Vs. Alpha	0.048860
	Omicron Vs. Alpha	0.100258
	Beta Vs. Beta	NA
	Delta Vs. Beta	0.021367
	Omicron Vs. Beta	0.072766
	Delta Vs. Delta	NA
	Omicron Vs. Delta	0.051399
	Omicron Vs. Omicron	NA
Oceania	Alpha Vs. Alpha	NA
	Beta Vs. Alpha	0.007550

	Delta Vs. Alpha	0.034721
	Omicron Vs. Alpha	0.110792
	Beta Vs. Beta	NA
	Delta Vs. Beta	0.027171
	Omicron Vs. Beta	0.103243
	Delta Vs. Delta	NA
	Omicron Vs. Delta	0.076072
	Omicron Vs. Omicron	NA

**Location(Country)**	**Comparison**	**Slope Value**
France	Alpha Vs. Alpha	NA
	Beta Vs. Alpha	0.007791
	Delta Vs. Alpha	0.035466
	Omicron Vs. Alpha	0.105387
	Beta Vs. Beta	NA
	Delta Vs. Beta	0.027675
	Omicron Vs. Beta	0.097596
	Delta Vs. Delta	NA
	Omicron Vs. Delta	0.069921
	Omicron Vs. Omicron	NA
Belgium	Alpha Vs. Alpha	NA
	Beta Vs. Alpha	0.014263
	Delta Vs. Alpha	0.033342
	Omicron Vs. Alpha	0.097112
	Beta Vs. Beta	NA
	Delta Vs. Beta	0.019079
	Omicron Vs. Beta	0.082849
	Delta Vs. Delta	NA
	Omicron Vs. Delta	0.063770
	Omicron Vs. Omicron	NA
Canada	Alpha Vs. Alpha	NA
	Beta Vs. Alpha	0.031635
	Delta Vs. Alpha	0.074619
	Omicron Vs. Alpha	0.143377
	Beta Vs. Beta	NA
	Delta Vs. Beta	0.042984
	Omicron Vs. Beta	0.111742
	Delta Vs. Delta	NA
	Omicron Vs. Delta	0.068758
	Omicron Vs. Omicron	NA
Africa	Alpha Vs. Alpha	NA
	Beta Vs. Alpha	0.010137
	Delta Vs. Alpha	0.040724
	Omicron Vs. Alpha	0.120853
	Beta Vs. Beta	NA

	Delta Vs. Beta	0.030587
	Omicron Vs. Beta	0.110715
	Delta Vs. Delta	NA
	Omicron Vs. Delta	0.080128
	Omicron Vs. Omicron	NA
Germany	Alpha Vs. Alpha	NA
	Beta Vs. Alpha	0.000393
	Delta Vs. Alpha	0.023663
	Omicron Vs. Alpha	0.095820
	Beta Vs. Beta	NA
	Delta Vs. Beta	0.023271
	Omicron Vs. Beta	0.095427
	Delta Vs. Delta	NA
	Omicron Vs. Delta	0.072157
	Omicron Vs. Omicron	NA
Ireland	Alpha Vs. Alpha	0.00
	Beta Vs. Alpha	0.020674
	Delta Vs. Alpha	0.060162
	Omicron Vs. Alpha	0.133592
	Beta Vs. Beta	0.00
	Delta Vs. Beta	0.039489
	Omicron Vs. Beta	0.112918
	Delta Vs. Delta	0.00
	Omicron Vs. Delta	0.073430
	Omicron Vs. Omicron	0.00
Israel	Alpha Vs. Alpha	0.00
	Beta Vs. Alpha	0.007646
	Delta Vs. Alpha	0.014176
	Omicron Vs. Alpha	0.089077
	Beta Vs. Beta	0.00
	Delta Vs. Beta	0.006530
	Omicron Vs. Beta	0.081432
	Delta Vs. Delta	0.00
	Omicron Vs. Delta	0.074902
	Omicron Vs. Omicron	0.00
Italy	Alpha Vs. Alpha	0.00
	Beta Vs. Alpha	0.009695
	Delta Vs. Alpha	0.025031
	Omicron Vs. Alpha	0.099349
	Beta Vs. Beta	0.00
	Delta Vs. Beta	0.015336
	Omicron Vs. Beta	0.089654
	Delta Vs. Delta	0.00
	Omicron Vs. Delta	0.074318
	Omicron Vs. Omicron	0.00

Japan	Alpha Vs. Alpha	0.00
	Beta Vs. Alpha	0.001599
	Delta Vs. Alpha	0.008899
	Omicron Vs. Alpha	0.058610
	Beta Vs. Beta	0.00
	Delta Vs. Beta	0.007301
	Omicron Vs. Beta	0.057012
	Delta Vs. Delta	0.00
	Omicron Vs. Delta	0.049711
	Omicron Vs. Omicron	NA
Netherlands	Alpha Vs. Alpha	NA
	Beta Vs. Alpha	0.012271
	Delta Vs. Alpha	0.051898
	Omicron Vs. Alpha	0.110896
	Beta Vs. Beta	NA
	Delta Vs. Beta	0.039627
	Omicron Vs. Beta	0.098625
	Delta Vs. Delta	NA
	Omicron Vs. Delta	0.058998
	Omicron Vs. Omicron	NA
Poland	Alpha Vs. Alpha	NA
	Beta Vs. Alpha	0.021080
	Delta Vs. Alpha	0.082703
	Omicron Vs. Alpha	0.135399
	Beta Vs. Beta	NA
	Delta Vs. Beta	0.061623
	Omicron Vs. Beta	0.114319
	Delta Vs. Delta	NA
	Omicron Vs. Delta	0.052696
	Omicron Vs. Omicron	NA
South Korea	Alpha Vs. Alpha	NA
	Beta Vs. Alpha	0.014668
	Delta Vs. Alpha	0.054380
	Omicron Vs. Alpha	0.080371
	Beta Vs. Beta	NA
	Delta Vs. Beta	0.039712
	Omicron Vs. Beta	0.065703
	Delta Vs. Delta	NA
	Omicron Vs. Delta	0.025991
	Omicron Vs. Omicron	NA
Spain	Alpha Vs. Alpha	NA
	Beta Vs. Alpha	0.073431
	Delta Vs. Alpha	0.083084
	Omicron Vs. Alpha	0.150894
	Beta Vs. Beta	NA

	Delta Vs. Beta	0.009653
	Omicron Vs. Beta	0.077463
	Delta Vs. Delta	NA
	Omicron Vs. Delta	0.067810
	Omicron Vs. Omicron	NA
Switzerland	Alpha Vs. Alpha	NA
	Beta Vs. Alpha	0.009731
	Delta Vs. Alpha	0.034113
	Omicron Vs. Alpha	0.105482
	Beta Vs. Beta	NA
	Delta Vs. Beta	0.024382
	Omicron Vs. Beta	0.095751
	Delta Vs. Delta	NA
	Omicron Vs. Delta	0.071369
	Omicron Vs. Omicron	NA
Turkey	Alpha Vs. Alpha	NA
	Beta Vs. Alpha	0.097631
	Delta Vs. Alpha	0.147481
	Omicron Vs. Alpha	0.200871
	Beta Vs. Beta	NA
	Delta Vs. Beta	0.049850
	Omicron Vs. Beta	0.103240
	Delta Vs. Delta	NA
	Omicron Vs. Delta	0.053390
	Omicron Vs. Omicron	NA
USA	Alpha Vs. Alpha	NA
	Beta Vs. Alpha	0.002825
	Delta Vs. Alpha	0.024760
	Omicron Vs. Alpha	0.109624
	Beta Vs. Beta	NA
	Delta Vs. Beta	0.021935
	Omicron Vs. Beta	0.106799
	Delta Vs. Delta	NA
	Omicron Vs. Delta	0.084864
	Omicron Vs. Omicron	NA
Mexico	Alpha Vs. Alpha	NA
	Beta Vs. Alpha	0.016381
	Delta Vs. Alpha	0.041005
	Omicron Vs. Alpha	0.111893
	Beta Vs. Beta	NA
	Delta Vs. Beta	0.024623
	Omicron Vs. Beta	0.095511
	Delta Vs. Delta	NA
	Omicron Vs. Delta	0.070888
	Omicron Vs. Omicron	NA
